# Reduced expression of FRG1 facilitates breast cancer progression via GM-CSF/MEK-ERK axis by abating FRG1 mediated transcriptional repression of GM-CSF

**DOI:** 10.1038/s41420-022-01240-w

**Published:** 2022-11-03

**Authors:** Bratati Mukherjee, Ankit Tiwari, Ananya Palo, Niharika Pattnaik, Subrat Samantara, Manjusha Dixit

**Affiliations:** 1 National Institute of Science Education and Research, School of Biological Sciences, Bhubaneswar, Odisha 752050 India; 2 Homi Bhabha National Institute, Training School Complex, Anushaktinagar, Mumbai, 400094 India; 3 AMRI Hospital, Bhubaneswar, 751030 Odisha India; 4 Acharya Harihar Regional Cancer Centre (AHRCC), Cuttack, 753007 Odisha India

**Keywords:** Breast cancer, Metastasis

## Abstract

Multiple molecular subtypes and distinct clinical outcomes in breast cancer, necessitate specific therapy. Moreover, despite the improvements in breast cancer therapy, it remains the fifth cause of cancer-related deaths, indicating the involvement of unknown genes. To identify novel contributors and molecular subtype independent therapeutic options, we report reduced expression of FRG1 in breast cancer patients, which regulates GM-CSF expression via direct binding to its promoter. Reduction in FRG1 expression enhanced EMT and increased cell proliferation, migration, and invasion, in breast cancer cell lines. Loss of FRG1 increased GM-CSF levels which activated MEK/ERK axis and prevented apoptosis by inhibiting p53 in an ERK-dependent manner. FRG1 depletion in the mouse model increased tumor volume, phospho-ERK, and EMT marker levels. The therapeutic potential of anti-GM-CSF therapy was evident by reduced tumor size, when tumors with decreased FRG1 were treated with anti-GM-CSF mAb. We found an inverse expression pattern of FRG1 and phospho-ERK levels in breast cancer patient tissues, corroborating the in vitro and mouse model-based findings. Our findings first time elucidate the role of FRG1 as a metastatic suppressor of breast cancer by regulating the GM-CSF/MEK-ERK axis.

## Introduction

Breast cancer remains at the top of cancer-related deaths among women, with a mortality rate of 6.9% [1]. Epithelial to mesenchymal (EMT) transition, which comprises the detachment of cancer cells from their primary origin, intravasation, and formation of metastatic growth at distant sites, accounts for majority of the deaths associated with breast cancer [2]. Even after the significant progress in primary breast cancer therapy, metastasis and recurrence are the major cause of reduced survival [3]. Finding out the role and mechanism of additional genes which affect EMT can provide additional opportunities to improve the survival rate.

FSHD region gene 1 (FRG1), which is mainly known for being the candidate gene for facioscapulohumeral muscular dystrophy (FSHD), has recently shown its potential as a tumor suppressor gene. Our earlier study first time reported the correlation of FRG1 downregulation with oral cavity, colorectal and gastric carcinoma [4]. Depletion of FRG1 levels increased cancerous properties of prostate cancer cell lines via activation of the p38-MAPK [5]. Mechanistically, reports have suggested FRG1’s involvement in pre-mRNA processing [6], F-actin-bundling [7], and angiogenesis [8]. The role of FRG1 in breast cancer is mostly unexplored. Expression profiling of the genes associated with triple-negative breast cancer (TNBC) using the MDA-MB-231 cell line showed FRG1 as one of the significantly downregulated genes which were connected with the migratory potential of breast cancer cells [9]. However, the exact biological function of FRG1 in breast cancer and its mechanism are entirely unknown.

So far, many cytokines such as IL6/8/10, TNFα, IFNγ, growth factors TGFβ, bFGF, and VEGF have been reported to involve with EMT in breast cancer [10]. Granulocyte-macrophage colony-stimulation factor (GM-CSF), a potent hematopoietic growth factor mainly known for its immunomodulatory function in the tumor niche, has recently been reported to play a role in EMT in colon cancer [11]. Elevated expression of GM-CSF is clinically correlated with advanced histological grade, metastasis, and poor prognosis in patients with prostate cancer, breast cancer [12], and pancreatic ductal carcinoma [13]. Nevertheless, very little is known about the upstream regulation and downstream signaling mechanism coordinating GM-CSF-mediated metastatic colonization. Previously we found enhanced GM-CSF expression in FRG1-depleted prostate cancer cells [5].

In the current study, we first report the FRG1-mediated regulation of GM-CSF in breast carcinoma. Prior to this, no information was available on the detailed mechanism of how GM-CSF promotes EMT in any cancer. Here we have shown that FRG1 binds to the GM-CSF promoter and inhibits its expression. The loss of FRG1 resulted in increased cell proliferation, migration, and invasion triggered by increased levels of GM-CSF and the activation of the MEK/ERK pathway, in both the cell lines of luminal (ER+; MCF7) and basal (TNBC; MDA-MB-231) origin. We have validated our in vitro findings in breast cancer patient tissues and mouse models. We have also shown the therapeutic potential of anti-GM-CSF antibody in the mouse model. Overall, here we report the role and molecular mechanism of a new gene, FRG1, in breast cancer, which has the potential to be explored as a therapeutic target irrespective of molecular subtypes.

## Result

### FRG1 affects the tumorigenic properties of breast cancer cells

**Fig. 1:**
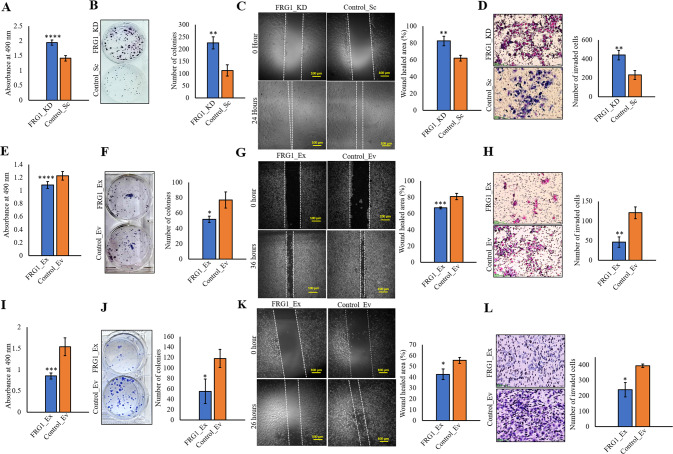
Effect of FRG1 alteration on tumorigenic properties of breast cancer cells of different molecular subtypes. The levels of FRG1 were modulated in two breast cancer cell lines of different molecular subtypes and subjected to various cell-based assays; **A**, **B** Representative images of MTS assay (**A**) and colony formation assay (**B**) showing the difference in proliferative property of MCF7 cells due to FRG1 knockdown (FRG1_KD vs. Control_Sc). OD values were taken at 490 nm at 24 h. Bar diagrams show the difference between the two groups. **C** Representative images of scratch wound-healing assay in MCF7 cells with depleted FRG1 (FRG1_KD) and corresponding control (Control_Sc). The bar graph depicts the difference in the percentage of wound closure between the two groups. Scale bar, 100 μm. **D** Representative images of matrigel transwell invasion assay in MCF7 cells with reduced FRG1 level (FRG1_KD) and respective control (Control_Sc). The bar graph depicts the difference in the number of invaded cells between the two groups. Scale bar, 50 μm. **E**, **F** Representative images of MTS assay (**E**) and colony formation assay (**F**) in MCF7 cells with an elevated level of FRG1 (MCF7_Ex) and control (Control_Ev). Bar graph showing the difference between the two groups. **G** Representative images of scratch wound-healing assay in MCF7_Ex and Control_Ev. Bar graph showing the difference in wound closure between the two groups. Scale bar, 100 μm. **H** Representative images of matrigel transwell invasion assay in MCF7_Ex and Control_Ev. The difference in invaded cells between the two groups is shown in the bar graph. Scale bar, 50 μm. **I**, **J** Representative images of MTS assay (**I**) and colony formation assay (**J**) in MDA-MB-231 cells with elevated expression of FRG1 (FRG1_Ex) and their control (Control_Ev). Bar graphs indicate the difference between the two groups. **K** Representative images showing scratch wound-healing assay performed in MDA-MB-231_FRG1_Ex and Control_Ev. The bar diagram shows the difference in wound healing percentage between the two groups. Scale bar, 100 μm. **L** Representative images of transwell invasion assay, performed in MDA-MB-231_Ex and Control_Ev. The bar diagram shows the difference in invaded cells between the FRG1_Ex and Control_Ev. Scale bar, 50 μm. Experiments were performed in triplicate, two-tailed unpaired student’s t-test was used to compare the two groups’ differences. Results are presented as mean ± SD. *P ≤ 0.05; **P ≤ 0.01; ***P ≤ 0.001; ****P ≤ 0.0001.

We prepared stable lines with FRG1 expression perturbation in estrogen receptor-positive (ER+) cells MCF7 which has moderate endogenous expression of FRG1 (Supplementary Fig. S1A). The reduction of FRG1 expression in MCF7 increased the proliferation rate in MTS and colony formation assays compared to the control group (Fig. 1A, B). Correspondingly, increased FRG1 expression decreased the rate of proliferation in both assays (Fig. 1E, F). To explore the metastatic potential, we checked the effect of FRG1 expression on cell migration. The wound-healing assay showed enhanced cell migration due to reduced FRG1 expression (Fig. 1C). Matrigel invasion assay corroborated these findings (Fig. 1D). We observed the opposite effect on wound healing and invasion of MCF7 cells with ectopic expression of FRG1 (Fig. 1G, H). Besides, we generated FRG1 knockout MCF7 cells (FRG1_KO) and checked its effect on the tumorigenic properties (Supplementary Fig. S1B, C). In accordance with our findings in the FRG1 knockdown group, we also observed increased cell proliferation (Supplementary Fig. S1B) and migration (Supplementary Fig. S1C) due to FRG1 knockout.

To determine if the effect of FRG1 is molecular subtype-specific, we ectopically expressed FRG1 in TNBC cell line MDA-MB-231, which has low endogenous FRG1 expression (Supplementary Fig. S1A). In agreement with our observation in MCF7_FRG1_Ex cells, we observed a reduction in cell proliferation (Fig. 1I, J), migration (Fig. 1K), and invasion (Fig. 1L). Together these data suggest FRG1 expression can modulate the tumorigenic properties of breast cancer cell lines.

### Reduction of FRG1 activates MEK/ERK signaling

**Fig. 2:**
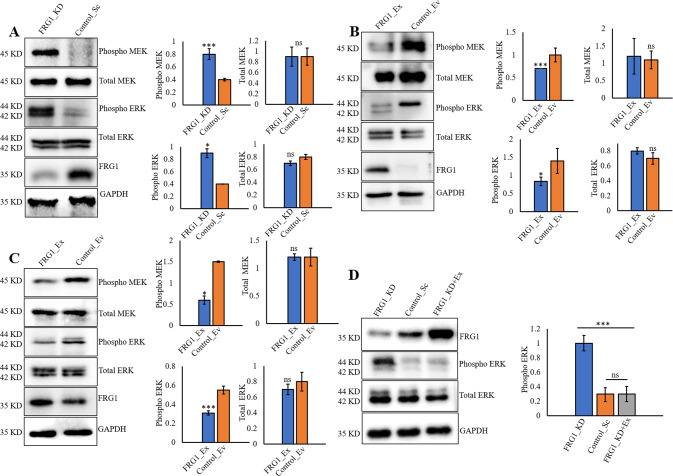
Effect of FRG1 expression modulation on MEK/ERK signaling axis. The expression of pERK and pMEK in MCF7 and MDA-MB-231 cells with altered FRG1 expression was examined by Western blots. **A**–**D** Representative Western blots and the densitometry-based bar graphs for **A** the difference in pERK and pMEK levels due to FRG1 knockdown (FRG1_KD vs. Control_Sc), **B** the difference in the activation of ERK and MEK in MCF7 cells with elevated FRG1 expression (FRG1_Ex vs. Control_Ev), **C** expression of pERK and pMEK in MDA-MB-231 cells ectopically expressing FRG1 (FRG1_Ex) and its control (Control_Ev), and **D** rescue of pERK levels in MCF7_KD cell transiently transfected with the FRG1 expression vector (FRG1_KD + Ex). Experiments were performed in triplicates. The intensity of the bands in each blot was measured by ImageJ software and normalized to GAPDH. Two-tailed unpaired student’s *t*-test was used to compare the difference between the two groups. Results are presented as mean ± SD. ns, *P* > 0.05, *P ≤ 0.05; ***P ≤ 0.001.

Intrigued by the effect of FRG1 expression level on the tumorigenic properties of breast cancer cells, we explored its effect on ERK and AKT, two frequently altered signaling pathways in cancers [14]. We observed that the reduced FRG1 level led to the activation of ERK and its upstream molecule MEK in MCF7 cells without the alteration in total-ERK or total-MEK levels (Fig. 2A). A similar trend was observed in FRG1 knockout MCF7 cells, where knockout of FRG1 led to increased expression of phospho-ERK (Supplementary Fig. S1D). On the other hand, ectopic expression of FRG1 decreased the activation of ERK and MEK in MCF7 (Fig. 2B) and MDA-MB-231 cells (Fig. 2C). As further corroboration of our findings, ectopic expression of FRG1 in MCF7_FRG1_KD cells nullified the change in phospho-ERK level (Fig. 2D).

Unexpectedly, we found that FRG1 depletion reduced AKT activation at the 308 position, and there was no effect at the 473 position in MCF7 cells (Supplementary Fig. S2A, B). Increased FRG1 expression also affected the activation of AKT only at the 308 position, in the opposite manner (Supplementary Fig. S2C, D). Previous studies have reported that activation in ERK signaling can suppress the AKT pathway [15]. We inhibited the ERK pathway in MCF7_FRG1_KD cells and found the reversal of the phospho-AKT 308 suppression (Supplementary Fig. S2E), which confirmed that inhibition of AKT activation was ERK-mediated. As expected, no effect of ERK inhibition was found on the level of phospho-AKT 473 (Supplementary Fig. S2E). This data suggests that reduced FRG1 expression might be involved in breast cancer progression by the activation of the ERK pathway.

### Depletion of FRG1 suppresses apoptosis by ERK-mediated inhibition of p53

**Fig. 3:**
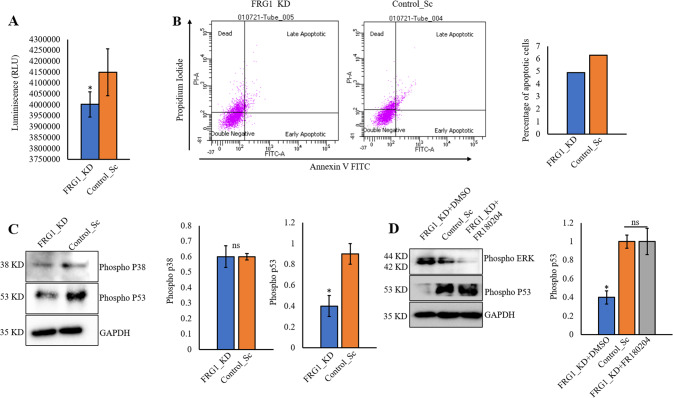
FRG1 depletion suppresses apoptosis in ERK-p53 dependent manner. The effect of FRG1 abrogation in apoptosis was assessed by caspase 3/7 assay and flow cytometry. The expression of major signaling molecules that control apoptosis was examined by immunoblotting. **A** Graphical representation depicts a change in luminescence level corresponding to caspase 3/7 in MCF7 cells with reduced FRG1 (FRG1_KD) and its control (Control_Sc) (*n* = 3). **B** Cell apoptosis was analyzed by flow cytometry using 488 nm excitation and 647 nm emission filters in MCF7_FRG1_KD and Control_Sc groups. The bar diagram shows the level of apoptotic cells in the MCF7_FRG1_KD and Control_Sc groups. (*n* = 1) **C** Effect of FRG1 expression depletion on p38 and p53 levels was assessed by immunoblotting in MCF7 cells (FRG1_KD vs. Control_Sc) (*n* = 3). **D** MCF7_FRG1_KD cells were treated with 10 μM of ERK inhibitor FR180204 (FRG1_KD + FR180204) and its solvent (FRG1_KD + DMSO) for 2 h; rescue in the activated ERK, and p53 levels was measured by Western blot (*n* = 2). The band intensity of each blot was measured by ImageJ and normalized to internal control GAPDH. In **A**, **C**, **D**, values are shown as mean ± SD. Two-tailed unpaired student’s *t*-test was used to compare the difference between the two groups. ns, *P* > 0.05, *P ≤ 0.05.

Loss of apoptotic control is a hallmark of cancer which permits cancer cells to survive longer and gives more time to accumulate the mutations [16]. Reduction in FRG1 level in MCF7 significantly decreased the downstream effector caspase 3/7 level (Fig. 3A). We made a coherent observation in a flow cytometry-based analysis of Annexin V/ propidium-iodide where FRG1 depletion reduced apoptosis in MCF7 cells (Fig. 3B). Mechanistically, we found that depletion of FRG1 reduced phospho-p53 level (Fig. 3C). No change in phospho-p38 level indicates reduced apoptosis in FRG1_KD cells may be independent of phospho-p38. (Fig. 3C). Several studies have reported ERK-mediated inhibition of p53 [17, 18]. To test this, we inhibited ERK and checked the levels of phospho-p53 in FRG1-depleted MCF7 cells. We observed restoration of phospho-p53 level in MCF7_FRG1_KD group treated with ERK inhibitor (Fig. 3D).

Overall, our findings suggest that a reduced level of FRG1 leads to an ERK-mediated decrease of phospho-p53, which reduces apoptosis.

### FRG1 depletion enhances the expression of EMT markers by activating MEK/ERK pathway

**Fig. 4:**
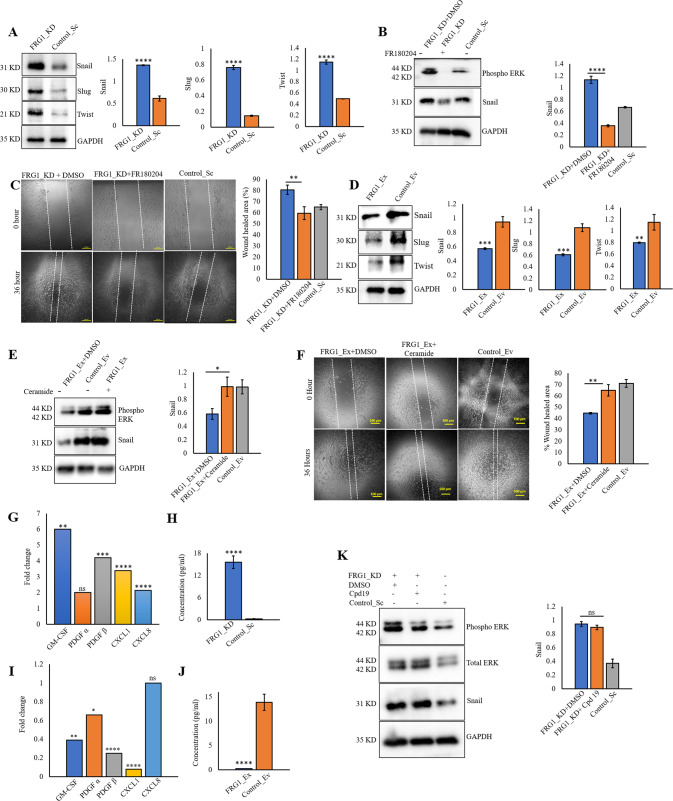
Reduction in FRG1 increases EMT via activation of the ERK pathway. FRG1 level was depleted in MCF7 (FRG1_KD) and elevated in MDA-MB-231 (FRG1_Ex) cells, and, subject to immunoblotting for EMT markers snail, slug, and twist to assess the effect of FRG1 expression modulation in EMT signaling. **A** Western blots show the effect of reduced FRG1 expression (FRG1_KD) on the levels of snail, slug, and twist. Corresponding bar graphs show the difference in expression between the FRG1_KD and Control_Sc groups. **B** MCF7_FRG1_KD cells were treated with 10 μM of ERK inhibitor FR180204 (FRG1_KD + FR180204) and DMSO (FRG1_KD + DMSO) for 2 h. The upper blot validates the reduction in the pERK level. Snail levels were examined in the FRG1_KD + FR180204 group compared to the FRG1_KD + DMSO group. **C** Representative images illustrate the difference in wound healing in the same set (as **B**). Scale bar, 100 μm. The bar graph shows the percentage of wound healing in MCF7_FRG1_KD + FR180204, MCF7_FRG1_KD + DMSO, and untreated Control_Sc groups. **D** Expression of EMT markers snail, slug, and twist were measured by immunoblots in MDA-MB-231 cells with ectopic expression of FRG1 (FRG1_Ex) versus control (Control_Ev). **E** MDA-MB-231 cells with elevated FRG1 level (FRG1_Ex) were treated with 5 μM of ERK activator Ceramide (FRG1_Ex + Ceramide) and DMSO (FRG1_Ex+DMSO) for 30 min. The uppermost blot validates the activation of ERK in MDA-MB-231_FRG1_Ex cells due to the administration of Ceramide. Rescue in snail level was measured by Western blot. **F** Rescue in cell migration was measured by wound-healing assay in MDA-MB-231_FRG1_Ex+Ceramide compared to MDA-MB-231_FRG1_Ex + DMSO group, along with Control_Sc. Scale bar, 100 μm. The bar diagram demonstrates the difference in the percentage of wound closure between MDA-MB-231_FRG1_Ex + DMSO and MDA-MB-231_FRG1_Ex + Ceramide groups. **G** Bar graph shows the effect of FRG1 reduction (FRG1_KD) on fold change in mRNA levels of GM-CSF, PDGFβ, PDGFα, CXCL1, and CXCL8 compared to Control_Sc as determined by qRT-PCR in MCF7 cells. **H** Bar graph of ELISA shows the difference in the level of GM-CSF due to FRG1 depletion (FRG1_KD) compared to the control (Control_Sc) in MCF7 cell line. **I** shows the effect of increased FRG1 expression (FRG1_Ex) in MDA-MB-231_Ex cells on the transcript levels of genes same as **G**. **J** Bar diagram depicts the difference in GM-CSF level due to ectopic expression of GM-CSF in MDA-MB-231 cell line. **K** MCF7_FRG1_KD cells were treated with CXCR2 inhibitor Cpd 19 (FRG1_KD + Cpd 19) and DMSO (FRG1_KD + DMSO) for 2 h. Western blots and graphical representation show its effect on the levels of pERK and snail in MCF7_FRG1_KD + DMSO, FRG1_KD + Cpd 19, and Control_Sc groups. GAPDH was used as internal control both for immunoblots and qRT-PCR. Shown results are representative of three independent experiments. Result values show mean ± SD value. Two-tailed unpaired student’s *t*-test was used to compare the difference between the two groups. ns, *P* > 0.05, *P ≤ 0.05; **P ≤ 0.01; ***P ≤ 0.001; ****P ≤ 0.0001.

It is well established that EMT increases cell migration, leading to metastasis in cancer [19]. As reduced FRG1 level increased the migration in cell-based assays, we checked its effect on EMT markers snail, slug, and twist, which have long been reported to trigger EMT through ERK signaling [20–22]. As hypothesized, we detected significant upregulation of snail, slug, and twist in MCF7_FRG1_KD cells (Fig. 4A). To validate, we inactivated the ERK pathway in MCF7_FRG1_KD cells and found abrogation of the ERK-mediated upregulation of EMT marker snail (Fig. 4B). Scratch wound-healing assay also revealed a similar effect (Fig. 4C). As expected, ectopic expression of FRG1 in MDA-MB-231 cells reduced the expression of EMT markers (Fig. 4D).

To further substantiate our findings, we treated MDA-MB-231_FRG1_Ex cells with ERK activator Ceramide, which restored the reduced level of phospho-ERK caused by ectopic expression of FRG1 (Fig. 4E). The same experimental setup also rescued snail expression levels (Fig. 4E) and cell migration (Fig. 4F). These findings also suggest that the effect of FRG1 on EMT is consistent in both the breast cancer cell lines. Our previous work [5] found that FRG1 perturbation changed the levels of inflammatory cytokines CXCL1, CXCL8, GM-CSF, PDGFα, and PDGFβ, which have been reported to affect EMT [11, 23, 24]. We found a significant increase in CXCL1, CXCL8, GM-CSF, PDGFα, and PDGFβ transcript levels due to FRG1 depletion (Fig. 4G) in MCF7 cells, the opposite effect was observed with the ectopic expression of FRG1 in MDA-MB-231 cells (Fig. 4I). Further analysis of the effect of altered FRG1 levels on GM-CSF concentration in the conditioned media harvested from MCF7 and MDA-MB-231 cells, confirmed our findings at protein levels (Fig. 4H, J). To ascertain the specificity of the observation, we inhibited the ERK pathway in MCF7_FRG1_KD cells, which counterbalanced the increase in CXCL1, CXCL8, PDGFα, and PDGFβ transcript levels caused by FRG1 depletion, suggesting that the effect was downstream of ERK (Supplementary Fig. S2F).

To elucidate if CXCL1 and CXCL8 were responsible for ERK activation and downstream change in EMT markers [25] we inhibited their receptor CXCR2 by using CXCR2 antagonist Cpd 19 [26]. We found no effect in phospho-ERK and snail levels (Fig. 4K), which suggests ERK activation is not downstream of CXCR2-CXCL1/8. Interestingly, we found that ERK pathway inhibition in MCF7_FRG1_KD could not nullify the increase in GM-CSF level caused by FRG1 reduction (Supplementary Fig. S2F), which implies that the effect on GM-CSF is upstream of ERK.

We propose that reduced FRG1 leads to ERK-mediated upregulation of cytokines except for GM-CSF, and FRG1 could act upstream of GM-CSF.

### Effect of FRG1 on ERK is GM-CSF mediated

**Fig. 5:**
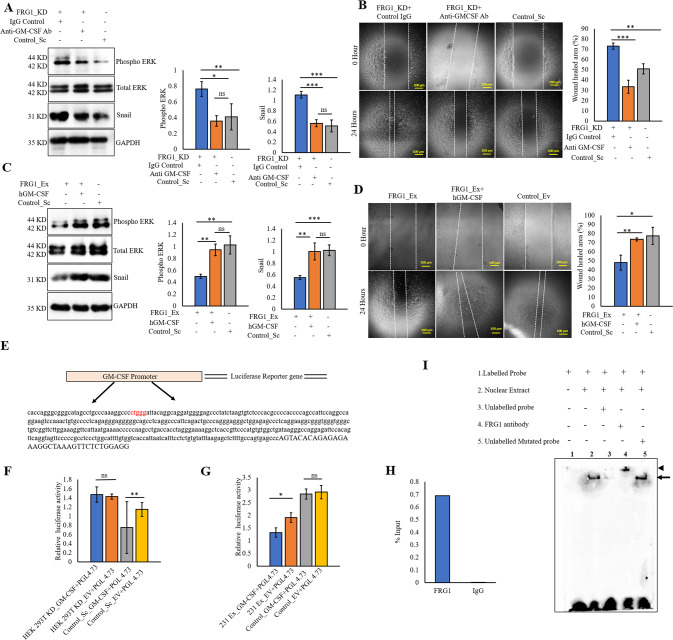
The effect of FRG1 on ERK activation and EMT markers is mediated by direct binding to the GM-CSF promoter and controlling its expression. **A**–**D** MCF7 cells with reduced FRG1 (FRG1_KD) and elevated FRG1 (FRG1_Ex) levels were treated with anti-GM-CSF antibody and human recombinant GM-CSF (hGM-CSF), respectively. The effect of these modulations was assessed by immunoblotting and wound-healing assay. **A** pERK, and snail expression was measured by Western blot in MCF7 cells with FRG1 knockdown treated with 2 mg/ml of anti-GM-CSF antibody (FRG1_KD + GM-CSF Ab) and control IgG (FRG1_KD + IgG) for an hour, along with untreated Control_Sc. (*n* = 3). **B** Representative images show cell migration in wound-healing assay in the same set (as **A**). The bar diagram depicts the difference in wound closure percentage among FRG1_KD + Control IgG, FRG1_KD + GM-CSF Ab, and untreated Control_Sc. Scale bar, 100 μm. (*n* = 3) **C** Rescue of pERK and snail levels was measured by Western blot in MCF7 cells with ectopic expression of FRG1, treated with 100 ng of human recombinant GM-CSF (FRG1_Ex + hGM-CSF) and PBS (FRG1_Ex+PBS) for 30 min. (*n* = 3). **D** Representative images showing the rescue in cell migration in the same set (as **C**) in wound-healing assay. The bar graph shows the difference in the percentage of wound healing among FRG1_Ex + hGM-CSF, FRG1_Ex + PBS, and untreated Control_Ev. Scale bar, 100 μm, *n* = 3. **E**–**I**, Luciferase assay, EMSA, and ChIP were carried out to investigate the binding potential of FRG1 on GM-CSF promoter. **E** Diagram shows the presence of CTGGG site on GM-CSF promoter, in 376 bp upstream of the transcription start site. **F** Bar diagram shows the difference in relative luciferase activity (firefly/renilla) using pGL4.23 vector with GM-CSF promoter and empty vector in one set of HEK 293 T cells with depleted FRG1 expression (HEK 293 T KD_GM-CSF + pGL4.73 vs. HEK 293 T KD_EV + pGL4.73). In another set same transfections were done in HEK 293 T cells with basal FRG1 expression (Control_Sc_GM-CSF + pGL4.73 vs. Control_Sc_EV + pGL4.73). **G** Bar diagram shows the difference in relative luciferase activity (firefly/renilla) using pGL4.23 vector with GM-CSF promoter and empty vector in one set of MDA-MB-231 cells with elevated FRG1 (231 Ex_GM-CSF + pGL4.73 vs. 231 Ex_EV + pGL4.73). In another set same transfections were done in MDA-MB-231 cells containing empty vectors (Control_GM-CSF + pGL4.73 vs. Control_EV + pGL4.73). **H** Graphical representation of ChIP assay, carried out in HEK 293 T cells. DNA fragments were immunoprecipitated with FRG1 antibody and IgG (negative control). Enrichment of the proteins on the GM-CSF promoter was calculated using the percent input (% input) method. **I** Representative image of EMSA confirms the binding of FRG1 on the GM-CSF promoter. The first lane shows a biotinylated probe only. The second lane shows a shift (arrow) in the mobility of the biotinylated probe incubated with nuclear protein extract from the HEK 293 T cell line. The third lane shows competitive binding between the excess amount of unlabeled probes and biotinylated probes with the nuclear protein extract. The fourth lane denotes the super shift (arrowhead) in the probe mobility due to binding with the FRG1 antibody. The fifth lane shows the shift (arrow) resulting from competitive binding between the excess amount of mutated unbiotinylated probe and biotinylated probe with the nuclear protein extract. Result values show mean ± SD value. Two-tailed unpaired student’s *t*-test was used to compare the difference between the two groups. ns, *P* > 0.05,*P ≤ 0.05; **P ≤ 0.01; ***P ≤ 0.001; ****P ≤ 0.0001.

So far, the role of GM-CSF in breast cancer has not been fully understood. We checked the effect of GM-CSF levels in MCF7 cells and found that GM-CSF enhanced the tumorigenic properties of MCF7 cells by upregulating cell proliferation and migration (Supplementary Fig. S3A, B). Also, ectopic administration of GM-CSF upregulated the expression of phospho-ERK and snail (Supplementary Fig. S3C). In order to confirm that GM-CSF is downstream of FRG1 and upstream of ERK, we perturbed GM-CSF levels in the cells with altered expression of FRG1. GM-CSF inhibition resulted in the downregulation of phospho-ERK and snail in MCF7_FRG1_KD cells (Fig. 5A), which eventually reduced cell migration in scratch wound-healing assay (Fig. 5B). Correspondingly, the opposite effect was observed in MCF7_FRG1_Ex cells upon treatment with exogenous human recombinant GM-CSF (Fig. 5C, D). These results suggest that the effect of FRG1 on the ERK pathway might be GM-CSF mediated.

### FRG1 binds to the GM-CSF promoter and regulates its expression

Based on the observations above, we hypothesized that FRG1 might be a direct transcriptional regulator of GM-CSF. Our previous work has shown “CTGGG” as a binding site for FRG1. We found six “CTGGG” sequences in the GM-CSF promoter region within 907 bp upstream of the transcription start site (Fig. 5E). Luciferase reporter assay using the promoter of GM-CSF (907 bp upstream of transcription start site in pGL4.23) revealed increased luciferase activity in HEK 293 T cells compared to the empty vector control (Fig. 5F). On the contrary, no change in luciferase activity was observed in HEK 293 T with FRG1 depletion, between the GM-CSF promoter and the control groups (Fig. 5F). When MDA-MB-231 cells with increased FRG1 expression levels were transfected with the same constructs, we found a substantial decrease in luciferase activity in cells containing GM-CSF promoter than control, while no change was observed in relative luciferase intensity in MDA-MB-231 (Control_Ev) cells with basal FRG1 expression between the GM-CSF promoter and the control groups (Fig. 5G). This observation strengthened that FRG1 more likely possessed an inhibitory effect on GM-CSF expression, which agrees with our previous observations.

Additionally, to confirm the binding of FRG1 on the GM-CSF promoter, a ChIP assay was performed in HEK 293 T cells. As shown in Fig. 5H, enrichment of FRG1 protein on GM-CSF promoter fragment was found after immunoprecipitation with anti FRG1 antibody but not by the negative control IgG. This approves our hypothesis that FRG1 binds to the GM-CSF promoter. To further validate, a competitive EMSA on labeled oligos was carried out with an increased amount of unlabeled oligos. The result showed that oligos were sufficient to compete for the binding. Thereby drastic reduction in the intensity of the shift was observed. Furthermore, when the binding complex was subjected to FRG1 specific antibody, a shift was noted, indicating the binding of FRG1 to the oligos (Fig. 5I). These results confirm in vitro binding of FRG1 on the CTGGG site of GM-CSF promoter.

Therefore, our data strongly support the notion that FRG1 hinders EMT progression in breast cancer by inhibiting GM-CSF-mediated ERK activation.

### Reduced expression of FRG1 in patient samples is inversely correlated with phospho-ERK expression

**Fig. 6:**
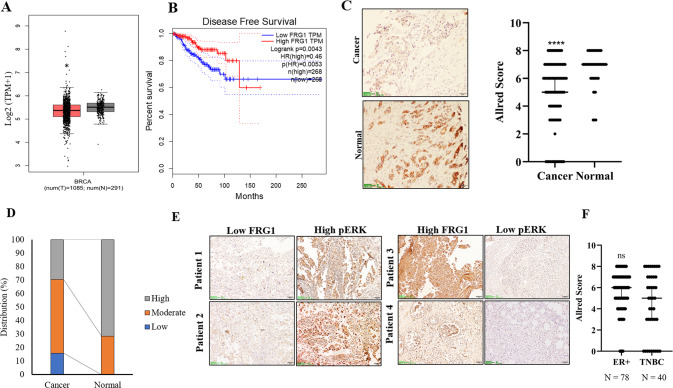
Low FRG1 is associated with breast carcinogenesis and elevated pERK expression in breast cancer patients. **A**, **B** Analysis of RNA sequencing data from TCGA and GTEx databases using GEPIA webserver. **A** Box plot shows the levels of FRG1 transcripts in cancer (*n* = 1085) and normal (*n* = 291) samples applying the default parameters *p* value cutoff 0.01, log_2_FC value cutoff 1, log scale, and jitter size 0.4. **B** shows the percentage of disease-free survival (RFS) and 95% confidence interval in the high FRG1 and low FRG1 expression groups. **C** Representative IHC images of FRG1 expression in human breast cancer and adjacent normal tissues. The Scatter plot shows the comparison of median Allred scores of FRG1 expression between breast cancer (*n* = 194) and adjacent normal (*n* = 56) tissues. The difference between the two groups in FRG1 expression was calculated using the Mann–Whitney *U*-test. **D** Graphical distribution depicts the frequency of cancer vs. normal tissues according to the Allred score of FRG1. The bar diagram shows the percent distribution of individuals having high, moderate, and low levels of FRG1 in breast cancer patients (*n* = 194) and normal tissue samples (*n* = 56). The X-axis represents two groups; cancer and adjacent normal. The Y-axis denotes the percentage of individuals having high, moderate, and low levels of FRG1. **E** Representative images of FRG1 and pERK expression levels detected in breast cancer patients using IHC (*n* = 10). **F** X-axis of the scatter plot shows median Allred scores for the FRG1 level in ER-positive (*n* = 78) and TNBC (*n* = 40) breast cancer patients. Y-axis denotes the Allred score for the FRG1 expression level. ns, *P* > 0.05, ****P ≤ 0.0001.

GEPIA-based analysis revealed lower expression of FRG1 transcripts in cancer patients (*n* = 1085) compared to normal (*n* = 291) (Fig. 6A) [27]. GEPIA also depicted that the patient group with a high level of FRG1 had a higher probability of disease-free survival (Fig. 6B). Kaplan–Meier plotter-based survival analysis also showed that breast cancer patients (containing wild-type p53) with a high level of FRG1 had a higher probability of recurrence-free survival (Supplementary Fig. S4A) [28].

To further authenticate TCGA dataset-based findings, we collected 194 breast cancer tissue samples with 56 normal adjacent tissues and performed IHC for FRG1 expression. We observed a significant downregulation of FRG1 levels in cancer patient samples compared to the normal tissue (Fig. 6C). Out of 194 cancer tissues, 57 indicated (29%) high FRG1 expression (AS: 7–8), 106 indicated (55%) moderate expression (AS: 3–6), and only 31 showed (16%) low level of FRG1 (AS: 1–2). In contrast, out of 56 normal tissues, 40 showed (71%) high FRG1 level (AS: 7–8), and 16 showed (29%) moderate FRG1 expression (AS: 3–6). No normal counterparts showed low FRG1 expression (AS: 1–2) (Fig. 6D). We observed an inverse relationship between FRG1 and phospho-ERK expression levels (*n* = 10) (Fig. 6E), strengthening our in vitro findings.

Estrogen signaling is crucial for breast carcinogenesis as it profoundly contributes to the proliferation of breast cancer cells. So, we checked whether the loss of FRG1 activated the ER signaling. To find out the correlation, we segregated breast cancer patient tissue samples according to their ER/PR/HER2 status and checked the expression of FRG1. We did not see any significant difference in FRG1 expression levels between ER + breast cancer tissues and TNBC patients (median FRG1 AS: 6 versus 5) (Fig. 6F). In MCF7 cells, we found upregulation in phospho-ER due to FRG1 depletion (Supplementary Fig. S4B), and precisely the opposite in the case of ectopic expression of FRG1 in MCF7 cells (Supplementary Fig. S4C). To examine whether ER activation was concomitant to ERK activation, we treated FRG1_KD MCF7 cells with ERK inhibitor FR180204 and found a significant reduction in phospho-ER levels. This suggests an ERK-mediated upregulation of ER in FRG1-depleted MCF7 cells (Supplementary Fig. S4D).

Taken together, our findings imply an inverse relation between FRG1 and ERK activation in breast cancer patients. As a downstream effect, FRG1 can affect ER activation in ERK-dependent manner.

### Loss of FRG1 promotes tumor development and metastasis in vivo via regulating GM-CSF/ERK

**Fig. 7:**
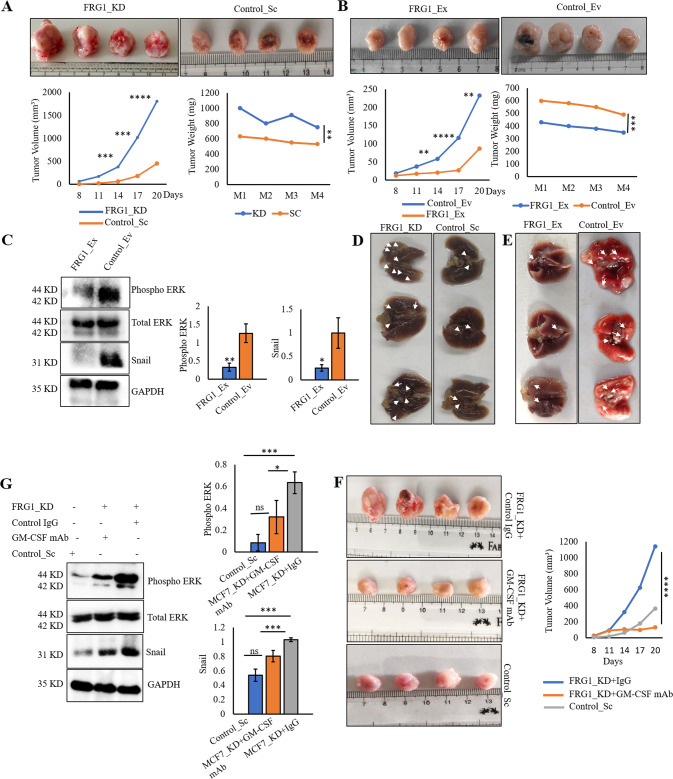
Reduced expression of FRG1 promotes tumor growth and metastasis in BALB/c mice. In vivo experiments were carried out to validate the tumorigenesis and metastatic potential of FRG1. **A**–**C** BALB/c mice were injected with 4T1 cells with reduced (4T1_FRG1_KD) and elevated levels of FRG1 (4T1_FRG1_Ex), along with controls (Control_Sc and Control_Ev, respectively). Tumor volume and weight were measured. Protein extracts were isolated from the tumors and subjected to immunoblots to observe the expression of pERK and snail. **A** Representative images of tumors in mice bearing 4T1_FRG1_KD cells and Control_Sc cells. A graphical representation of the same depicts the change in tumor volume and weight between the two groups (*n* = 4). **B** Representative images of tumors in mice bearing 4T1_FRG1_Ex cells and Control_Ev cells. A graphical representation of the same depicts the change in tumor volume and weight between the two groups (*n* = 4). **C** Tumor harvested from 4T1_FRG1_Ex, and Control_Ev mice show expression of pERK and EMT marker snail. **D**, **E** 4T1 cells with altered FRG1 levels were injected in the tail vein of BALB/c mice. After that, nodules in the lungs were counted. Representative images of the lung nodules as observed in mice bearing 4T1_FRG1_Ex (**D**) and 4T1_FRG1_KD (**E**) cells with their respective controls. **F**, **G** BALB/c mice were injected with 4T1_FRG1_KD cells to develop tumors and the corresponding control (Control_Sc). After 7 days, 4T1_FRG1_KD group mice were treated with anti-GM-CSF antibody (*n* = 4) or control_IgG (*n* = 4), till day 21. **F** Images of tumors harvested from the three groups (FRG1_KD + Control IgG, FRG1_KD + GM-CSF mAb, and Control_Sc). The graph shows tumor volume measured on different days. **G** Protein extracts were isolated from the three sets and subjected to immunoblotting to observe pERK and snail expression. Bar graphs show the difference in expression levels of pERK and snail levels among the three groups (*n* = 3). Immunoblots are showing the expression of pERK, with the GAPDH as a loading control. Results are presented as mean ± SD. Two-tailed unpaired student’s *t*-test was used to compare the difference between the two groups. ns, *P* > 0.05, *P ≤ 0.05; **P ≤ 0.01; ***P ≤ 0.001; ****P ≤ 0.0001.

To demonstrate the impact of FRG1 expression levels in vivo, we developed an orthotopic mice model by implanting 4T1 cells with depleted and elevated levels of FRG1, in the mammary fat pad of BALB/c mice. FRG1 knockdown significantly increased tumor volume and weight (Fig. 7A and Supplementary Fig. S5A). Correspondingly, ectopic expression of FRG1 significantly reduced tumor volume and weight (Fig. 7B and Supplementary Fig. S5B). Parallel to our cell line-based data, we found a reduced expression of phospho-ERK and snail in the FRG1 over-expression group (Fig. 7C). To assess its metastasis potential, we injected FRG1-depleted 4T1 cells in the tail vein of BALB/c mice. We observed more metastatic nodules in the lungs of the FRG1_KD group than in control (Fig. 7D). An opposite trend was identified in the set with FRG1 over-expression (Fig. 7E). These results corroborate our in vitro observations in the mouse model.

**Fig. 8:**
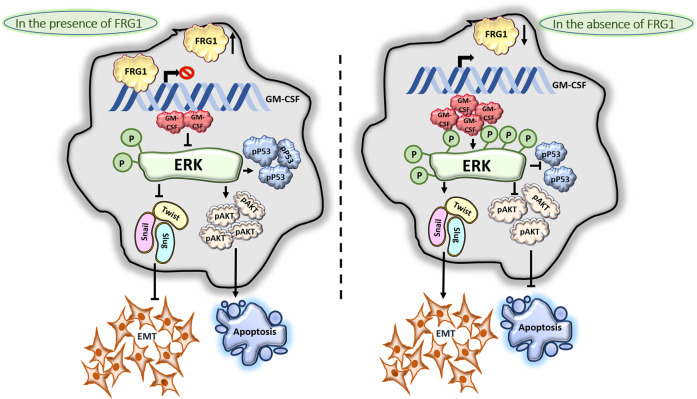
Schematic showing direct transcriptional regulation of FRG1 in suppressing GM-CSF mediated breast carcinogenesis. Left side of the illustration describes FRG1-mediated transcriptional repression of GM-CSF. FRG1 binds to the GM-CSF promoter and suppresses its transcription. In the presence of an optimal amount of FRG1 and lesser GM-CSF, activation of ERK gets reduced, which in turn downregulates snail, slug, and twist mediated EMT. On the other hand, a depleted level of pERK promotes the activation of AKT and p53 that facilitates apoptosis of the cells. Thus, the presence of FRG1 protects cells from GM-CSF-mediated tumorigenesis. Right panel shows the opposite effect that occurs due to a depleted level of FRG1. FRG1 downregulation leads to the upregulation of pERK. An increased amount of activated ERK inhibits the expression of pAKT and pP53 simultaneously, which in turn, inhibits cell apoptosis. Also, the upregulation of pERK increases the expression of EMT markers that leads cells toward EMT. Thus our model demonstrates FRG1-mediated suppression of breast carcinoma via GM-CSF/MEK/ERK signaling.

### Anti-GM-CSF treatment abrogates FRG1 depletion-based tumors growth in mice

To investigate the therapeutic potential of anti-GM-CSF therapy in breast cancer patients with reduced FRG1 expression, we did a mouse model-based study. We injected 4T1_FRG1_KD and Control_Sc cells subcutaneously into mice (*n* = 4). After the tumor reached a palpable size (7 days), one set of 4T1_FRG1_KD mice (*n* = 4) was intraperitoneally administrated with anti-GM-CSF neutralizing monoclonal antibody (mAb) (10 mg/kg body weight), and the other set of 4T1_FRG1_KD mice (*n* = 4) was injected with control IgG antibody (10 mg/kg body weight) alternative days till day 21. We found a significant reduction in tumor size in the set treated with GM-CSF mAb compared to IgG (Fig. 7F and Supplementary Fig. S5C). In support of our in vitro data, we also observed a reduction in phospho-ERK and snail in GM-CSF treated group (Fig. 7G). Henceforth our in vivo findings establish the role of FRG1-mediated regulation of GM-CSF. It also indicates that loss of FRG1, disrupts the suppression of GM-CSF, which might promote the proliferation and EMT by activating ERK and its downstream targets.

## Discussion

Metastatic dissemination in breast cancer accounts for 90% of cancer-related deaths [29]. Therefore, elucidating the molecular mechanism underlying the metastatic process has attracted considerable attention from researchers. Loss of function of tumor suppressor genes is often associated with increased metastasis and poor patient survival. In the case of breast cancer, different molecular subtypes represent discrete clinical outcomes. Hence, the search for novel targets that can act independently of molecular subtypes, will contribute to the development of more effective therapy.

Since the discovery of the FRG1 in 1996, most of the reports have highlighted its involvement in FSHD pathophysiology, muscle development, actin-bundling, and angiogenesis. Very few reports have shown an indirect association of FRG1 with cancer. Whole-exome sequencing identified some deleterious mutations in the FRG1 gene in calcifying fibrous tumor of the pleura [30]. Another whole-exome sequencing study, done in six follicular thyroid cell lines, reported a mutation in the FRG1 gene in cancer cell lines [31]. Our group showed FRG1-mediated activation of the p38-MAPK pathway in prostate cancer [5]. In the current study, our findings first-ever report the inverse association of FRG1 expression levels with breast cancer and unravel the underlying molecular mechanism using multiple model systems. Activation of ERK plays a pivotal role in cell proliferation, angiogenesis, and malignant transformation [32]. It supports tumor growth and cancer progression by upregulating various EMT-inducing factors [33]. TNBC, which is considered more aggressive and therapeutically challenging [34], is often associated with shorter patient survival with high expression of ERK [35]. The cross-talk between ERK and ERα signaling in luminal carcinoma may lead the cells toward chemoresistance [36]. In our case, we have found that reduced FRG1 expression led to the activation of ERK in both the breast cancer cell lines. We have further observed ERK-mediated elevated expression of the EMT markers snail, slug, and twist, which is consistent both in vitro and in vivo. Our findings may create enormous scope for research to develop novel therapeutics that can target upstream regulators of ERK and act irrespective of molecular subtypes of breast cancer.

The cross-talk between ERK and AKT molecules is an important determinant of the cell fate towards survival or apoptosis [18]. Surprisingly, although there was no change in AKT 473 activation, the depletion of FRG1 reduced the activation of AKT 308. Previous studies have shown ERK-mediated suppression of AKT activation [37], as was the case in our study, where the suppression of AKT 308 activation was rescued upon ERK inhibition in MCF7 cells. This observation supports the previous finding that MEK downregulation decreases AKT activation in EGFR and HER2-driven breast cancer [15]. Also, the activation of AKT was found to protect HeLa cells from apoptosis [38]. In this context, several reports suggest multiple mechanisms for MEK/ERK-mediated downregulation of AKT activation [15, 38, 39]. ERK has been shown to negatively regulate Gab1-PI3K binding, reducing the downstream AKT signaling [39], [15]. Also, inhibition of ERK, reduces EGFR phosphorylation, resulting in augmented AKT phosphorylation [40].

In addition to AKT-P38-P53 mediated activation of apoptosis, several studies have reported ERK-mediated regulation of p53 and apoptosis [41]. Inactivation of ERK promoted translocation of apoptosis-inducing factor in the nucleus, thus, causing apoptosis [18]. Another report suggests ERK promotes increased MDM2 expression and thus promotes the degradation of p53 [17]. We found reduced expression of phospho-p53 in FRG1-depleted MCF7 cells, which was rescued by the inhibition of ERK. This is in parallel to the literature that supports the MEK/ERK pathway can downregulate the activation of p53 and promote cell survival [18]. We put forward our hypothesis that FRG1 depletion activates the ERK pathway, which attenuates AKT and p53 phosphorylation that results in ERK-dependent inhibition of the apoptotic pathway.

Previously it was reported that GM-CSF promotes breast cancer pathogenesis by recruiting CCL-18+ macrophages into the tumor microenvironment [12]. Increased GM-CSF level in breast cancer is correlated with increased metastasis and poor patient survival [42]. Higher expression of GM-CSF receptors on nonhematopoietic cells in multiple tumor types has suggested its potential pro-tumorigenic factor, which is yet to be validated [43]. Expression of GM-CSF in skin carcinoma cells enhanced the metastatic growth and proliferation of cancer cells [44]. Also, in the head and neck [45], glioma [46], and osteosarcoma [47], autocrine stimulation of GM-CSF is reported to promote tumor growth. Although several reports have shown altered GM-CSF levels in multiple cancers, the underlying molecular mechanism in GM-CSF-mediated metastasis needs to be further addressed. The effect of the GM-CSF on the MAPK/ERK/ZEB1 pathway has been reported in colon cancer [11], but the detailed mechanism remains unexplored. The present study first time, discovered the in-depth role of GM-CSF in breast cancer and found that FRG1 acts as its repressor. When the expression of FRG1 is less in the cells, it leads to the expression of more GM-CSF, which in turn activates ERK-mediated EMT. Our findings have provided many missing links from FRG1 to GM-CSF to ERK to EMT. Besides, we have observed treatment with anti-GM-CSF mAb reduced tumor volume and EMT marker in the mouse model. Collectively, our study highlights the therapeutic potential of anti-GM-CSF therapy in cancer samples with low FRG1 expression.

To validate our findings, we performed a retrospective study in clinical patient samples and found reduced FRG1 expression in 71% of breast cancer patients' tissues. Reports suggest expression of ER in breast cancer is associated with favorable clinical outcomes [48]. We observed that in ER+ and TNBC samples, there was no difference in FRG1 expression levels. It emphasizes that the role of FRG1 in breast cancer is not subtype-specific. Yet, our in vitro findings suggest activation of ER in FRG1-depleted MCF7 cell line in an ERK-dependent manner showing cross-talk between FRG1-mediated regulation of ERK and ER signaling. Taken together the in vitro and in vivo data, the therapeutic potential of FRG1-mediated signaling can be explored in all molecular subtypes of breast cancer. Furthermore, survival analysis in GEPIA and Kaplan–Meier plotter designates the association of high FRG1 level with a higher recurrence-free survival rate in breast cancer patients, indicating a favorable role of FRG1 towards prognosis determination. Observation in large and stage-specific cohorts can confirm the authenticity of this observation.

In conclusion, reduced expression of FRG1 in breast cancer leads to transcriptional activation of GM-CSF, which promotes activation of ERK and EMT (Fig. 8). Identification of GM-CSF as an activator of ERK, and the cross-talk between AKT and ERK, can help in the development of a more efficient therapeutic strategy.

## Materials and methods

### Breast cancer patient sample collection

Our study included 194 breast cancer tissue samples with 56 adjacent normal tissues. From SRL diagnostics-Bhubaneswar, Apollo Hospitals-Bhubaneswar, and AHRCC-Cuttack, between 2014 to 2019, we collected tissues from 104 breast cancer patients, out of which 46 had the adjacent normal tissues. The patients who did not undergo any prior treatment before the surgery with known ER/PR/HER2 status, stage, and grade information as per the TNM system, were only included in the study. This study was approved by the Institutional Ethics Committee of NISER (NISER/IEC/2021-02) and AHRCC. For prospective sample collection, written consent was taken; for retrospective tissue FFPE blocks, the ethics committee waived the requirement of written consent. Additionally, a tissue microarray containing 90 tumors and 10 normal tissue samples was purchased (Biomax, MD, USA, #BC081120c).

### Immunohistochemistry (IHC)

From FFPE blocks, 5 µm thin sections were cut and deparaffinized by heating at 80 °C dry bath for an hour, then submerging the slides into xylene. Sections were rehydrated by a gradient of ethanol-100, 90, 70, 50%, and water. Heat-induced antigen retrieval was done in EnVision FLEX target retrieval solution (pH 9) high pH citrate buffer (DAKO, MN, USA) and blocked with EnVision FLEX Peroxidase blocking buffer (DAKO). Sections were incubated with primary and EnVision FLEX HRP secondary antibody (DAKO) for 2 hours and 30 minutes. We stained slides with EnVision FLEX DAB + Chromogen (DAKO) and counterstained them with Haematoxylin (Himedia, Mumbai, India). FRG1 expression was calculated using the Allred score (AS), as described previously [4]. Briefly, the Allred score was measured by combining the staining intensity of FRG1 protein in the cytoplasm and the percentage of cells stained positive for FRG1. Measurements of ‘staining intensity’ were categorized as weak “0–2”, moderate “3–6”, and strong “7–8”. FRG1 positive tumor tissue’s staining percentage were scored as “0” if 0%, “1” if 1%, “2” if 2–10%, “3” if 11–33%, “4” if 34–66% and “5” if ≥67%. Each sample was compared to the adjacent uninvolved tissue (if found) as a control.

### Cell culture, plasmids, generation of stable cell lines, and cell-based assays

Detailed methodology is provided in the supplementary information.

### RNA extraction and quantitative real-time PCR

According to the manufacturer’s protocol, total RNA was extracted from the cells using an RNeasy mini kit (Qiagen, Hilden, Germany). Reverse transcription was performed with 1 µg of RNA using the verso cDNA synthesis kit (Thermo Scientific, Lithuania). Each qPCR reaction was carried out using 10 ng of cDNA, 2x SYBR Green PCR Master Mix (Thermo Fisher, CA, USA), and respective primers (Supplementary Table 1) in Applied Biosystem 7500 system (Thermo Fisher, CA, USA). All reactions were done in triplicate. GAPDH was used as an internal control. The relative expression of each transcript was calculated using the ΔΔCt method.

### Enzyme-linked Immunosorbent assay (ELISA)

ELISA was performed to quantify the level of GM-CSF present in MCF7 and MDA-MB-231 cells with altered FRG1 expression. In total, 1 × 10^6^ cells were plated in a 100 mm culture dish in their respective culture media. After 12 h, the media was replaced by the media containing 2% FBS. After 3 days of incubation, the media was collected and centrifuged at 4000 rpm (4 °C) for 10 min to get rid of the cellular debris. The supernatant was aliquoted and stored at −80 °C till further use. 100 μl of this supernatant was used to carry out the ELISA using Human GM-CSF Quantikine ELISA Kit (R&D Systems, MN, USA) according to the manufacturer’s protocol. OD values were taken at 450 nm and 540 nm in the Varioscan multimode microplate reader (Thermo). During the final analysis, values obtained at 450 nm was subtracted from the values at 540 nm.

### Western blot

Cell lysates were prepared in ice-cold RIPA buffer (Thermo Scientific, IL, USA), supplemented with protease-phosphatase inhibitor (Thermo Scientific, IL, USA). Protein was quantified by the BCA protein estimation kit (Thermo Scientific, IL, USA). About 30–40 µg of protein samples were resolved on 12% sodium dodecyl sulfate-polyacrylamide gel electrophoresis (SDS-PAGE) and transferred onto a PVDF membrane (Millipore, Bangalore, India). The membrane was blocked using 5% BSA (MP Biomedicals, OH, USA) for an hour and incubated overnight with the primary antibodies (Supplementary Table 2). Blots were washed in 1X TBST buffer and detected with respective horseradish peroxidase (HRP) conjugated secondary antibodies (Abgenex, Bhubaneswar, India). The chemiluminescence signal was developed by SuperSignalTM West Femto maximum sensitivity substrate (Thermo Scientific, IL, USA) and detected in Chemidoc XRS + (Bio-Rad, CA, USA). Images were analyzed in ImageJ (NIH, MD, USA) software. All the experiments were performed in triplicate.

### Pharmacological compounds for inhibition and activation assay

Details are given in the supplementary information.

### Caspase 3/7 assay

Caspase 3/7 activity was measured using the caspase-Glo 3/7 assay kit (Promega, WI, USA) as per the manufacturer’s instructions. Briefly, 1 × 10^3^ cells were plated in a 96-well plate. After 24 h, 100 µl of caspase-Glo 3/7 reagent was added to each well and incubated for 20 min. Luminescence readings were measured in a Varioscan multimode microplate reader (Thermo Scientific, USA).

### Apoptosis assay

The cellular apoptosis was measured in FRG1-depleted MCF7 cells using Annexin FITC and PI staining kit (BD Pharmingen™, NJ, USA) following the manufacturer’s protocol, in FACS caliber (BD Biosciences, CA, USA). Flow cytometric data were analyzed by CellQuest Pro software (BD Biosciences).

### Luciferase reporter assay

GM-CSF promoter (accession no. P04141) was cloned into the pGL4.23 [luc2/minP] (Promega, WI, USA) vector. Appropriate cells were plated and transfected with 990 ng of pGL4.23_GM-CSF promoter construct and 10 ng internal control pGL4.74 [hRluc/TK] (Promega, WI, USA) using Lipofectamine 3000 (Invitrogen). After 48 h, cells were harvested using the lysis buffer provided with the Dual-Glo Luciferase assay kit (Promega, WI, USA). Firefly and renilla luminescence signal was quantified using Varioscan multimode microplate reader (Thermo Scientific). For each sample, firefly luciferase activity was normalized to renilla luciferase activity.

### Electrophoretic mobility shift assay (EMSA)

Nuclear extract was prepared from HEK 293 T cells expressing FRG1 using the NE-PER kit (Thermo Scientific, IL, USA). Oligonucleotides were commercially procured (IDT, IA, USA). The binding of FRG1 on the CTGGG site of the GM-CSF promoter was investigated using 3 fmol of biotinylated double-stranded oligonucleotides. Competitive EMSA was carried out using 3 pmol of unlabeled oligonucleotides. For the supershift assay, 1.5 µg of FRG1 antibody (Abcam, Cambridge, UK) was used following standard reaction conditions per the manufacturer’s protocol. Protein-DNA complexes were separated on 10% native polyacrylamide gels in 0.5X TBE, transferred on nylon membrane (Thermo Scientific, IL, USA), and crosslinked upon UV exposure for 5 min. Signal development was done using Chemiluminescent Nucleic Acid Detection Module Kit (Thermo Scientific) and detected in the Chemidoc XRS + system (Bio-Rad).

### Chromatin immunoprecipitation (ChIP) assay

ChIP assay was carried out with the chromatin (1 × 10^6^ cells) harvested from HEK 293 T cell line. Afterward, cells were processed using the ChIP kit (Abcam, USA) according to the manufacturer’s protocol. Briefly, first cells were washed using 1x PBS followed by cross-linking with 1.1% formaldehyde and Buffer A for 10 min at room temperature. Next, quenching of the cross-linking process was done using 1.5 M glycine for 5 min provided with the kit. The cell pellet was collected after centrifugation for 5 min at 4 °c. The cell pellet was lysed and then subjected to sonication to attain 200–600 bp fragments using a sonicator (Cole-Parmer, IL, USA) at maximum speed (30 times) for 35 s. Sheared chromatin was availed after centrifugation at 14,000 × *g* for 5 min. Immunoprecipitation was done by incubating the chromatin with the antibodies against FRG1 and negative control IgG at 4 °c overnight. After de-cross-linking and proteinase K treatment, immunoprecipitated chromatin was isolated and subjected to qRT PCR using the primers given in supplementary table 1.

### Differential expression and survival analysis

GEPIA webserver (http://gepia.cancer-pku.cn/about.html) was used to ascertain the differential FRG1 transcript level (cancer patients vs. TCGA normal and GTEx, rest of the default parameters- *p* value cutoff 0.01, log_2_FC value cutoff 1, log scale, and jitter size 0.4) and disease-free survival (DFS) (group cutoff quartile) in BRCA dataset (accessed on 24 December 2021).

We did relapse-free survival (RFS) analysis in Kaplan–Meier plotter (https://kmplot.com/analysis/, accessed on 16 Feb 2022) using mRNA gene chip data and auto-select the best cutoff in breast cancer patients with wild-type p53. Hazard ratio (HR) with a 95% of confidence interval (CI), and statistical significance were determined using log-rank test.

### Animal model-based assays

All animal experiments were performed following the protocol approved by the Institutional Animal Ethics Committee, NISER (NISER/SBS/IAEC/AH-109). Details of tumor development, metastasis, and in vivo GM-CSF inhibition experiments are given in the supplementary information.

### Statistical analysis

Statistical analysis was performed using GraphPad Prism 6.0 version (GraphPad Software Inc., CA, USA) and Microsoft Excel (Microsoft, WA, USA). To determine the statistical significance of the difference in mean values, a two-tailed unpaired Student’s *t*-test was applied. Mann–Whitney *U*-test was used to measure the correlation between Allred scores for FRG1 expression in cancer and normal samples. Data were presented as mean ± SD. *P* value ≤0.05 was considered to be significant.

## Supplementary information

Consent for authorship changes

Author Contribution Statement

Supplementary figures

Supplementary Materials and methods

Supplementary Table 1

Supplementary Table 2

Original Data File

## Data Availability

All the data generated and analyzed in the study are included in the article and its supplementary files.
