# Supplementary material for: Reduced expression of FRG1 facilitates breast cancer progression via GM-CSF/MEK-ERK axis by abating FRG1 mediated transcriptional repression of GM-CSF
Source: Cell Death Discov. 2022 Nov 3;8:442. doi: 10.1038/s41420-022-01240-w (PMC9633810; doi:10.1038/s41420-022-01240-w)
Supplement: Supplementary file 1 — Consent for authorship changes [file 41420_2022_1240_MOESM1_ESM.pdf]

Manjusha Dixit <manjusha@niser.ac.in>

Consent for the change in authorship

6 messages

Manjusha Dixit <manjusha@niser.ac.in> Thu, Oct 20, 2022 at 12:23 PM
To: Bratati Mukherjee <bratati.mukherjee@niser.ac.in>, Ankit Tiwari <ankittiwariacad2017@gmail.com>, Ananya Palo <ananya.palo@niser.ac.in>, NIHARIKA PATTNAIK <debnit2005@yahoo.co.in>, subrat samantara <subratsamantara@gmail.com>

Dear all,
The updated author list of our manuscript ‘Reduced expression of FRG1 facilitates breast cancer progression via GM-CSF/MEK-ERK axis by abating FRG1 mediated transcriptional repression of GM-CSF’ is now: Bratati Mukherjee, Ankit Tiwari, Ananya Palo, Niharika Pattnaik, Subrat Samantara, Manjusha Dixit.
Earlier it was "Bratati Mukherjee, Ankit Tiwari, Ananya Palo, Niharika Pattnaik, Tathagata Mukherjee, Subhasis Chattopadhyay, Subrat Samantara, Manjusha Dixit."
As per the reviewers comment, we removed the data included in " Figure 7H-K" which was about "Reduced FRG1 level may promote immune suppression". This data was contributed by "Tathagata Mukherjee and Subhasis Chattopadhyay".
If you agree with this authorship change, please reply to this mail "I agree".
Thank you,
Best regards,
Manjusha

--
Dr. Manjusha Dixit
Associate Professor
Room No. 204, School of Biological Sciences,
National Institute of Science Education and Research, Bhubaneswar
PO: Jatani, Khurda 752050, Odisha, India
Phone: Office 91-674 2494195, Lab 91-674-2494196

SCIENTIFIC REPORTS Editorial Board Member for Scientific Reports nature.com/scientificreports
Editorial Board Member for BMC Cancer bmccancer.biomedcentral.com BMC Part of Springer Nature

Ananya Palo <ananya.palo@niser.ac.in> Thu, Oct 20, 2022 at 12:49 PM
To: Manjusha Dixit <manjusha@niser.ac.in>

Dear ma'am,
I agree for the change in authorship.

Regards
[Quoted text hidden]

Bratati Mukherjee <bratati.mukherjee@niser.ac.in> Thu, Oct 20, 2022 at 12:56 PM
To: Manjusha Dixit <manjusha@niser.ac.in>

I agree with the authorship change.

Bratati Mukherjee,
Ph.D. Scholar, M.D Lab,
Cancer and Angiogenesis Research Group,
National Institute of Science Education and Research
(Dept. of Atomic Energy, India),
Bhubaneswar- 752050,
Contact No: 9348346566.

[Quoted text hidden]

---

subrat samantara <subratsamantara@gmail.com> Thu, Oct 20, 2022 at 7:56 PM
To: Manjusha Dixit <manjusha@niser.ac.in>

I agree
[Quoted text hidden]

NIHARIKA PATTNAIK <debnit2005@yahoo.co.in> Thu, Oct 20, 2022 at 8:23 PM
Reply-To: NIHARIKA PATTNAIK <debnit2005@yahoo.co.in>
To: manjusha@niser.ac.in, Bratati Mukherjee <bratati.mukherjee@niser.ac.in>, Ankit Tiwari <ankittiwariacad2017@gmail.com>, Ananya Palo <ananya.palo@niser.ac.in>, subrat samantara <subratsamantara@gmail.com>

I agree
Dr Niharika Pattnaik

Sent from Yahoo Mail on Android
[Quoted text hidden]

Ankit Tiwari <ankittiwariacad2017@gmail.com> Fri, Oct 21, 2022 at 3:56 PM
To: Manjusha Dixit <manjusha@niser.ac.in>

I agree.

Ankit
[Quoted text hidden]
