# Supplementary material for: Reduced expression of FRG1 facilitates breast cancer progression via GM-CSF/MEK-ERK axis by abating FRG1 mediated transcriptional repression of GM-CSF
Source: Cell Death Discov. 2022 Nov 3;8:442. doi: 10.1038/s41420-022-01240-w (PMC9633810; doi:10.1038/s41420-022-01240-w)
Supplement: Supplementary file 2 — Author Contribution Statement [file 41420_2022_1240_MOESM2_ESM.docx]

**Author Contributions:**

**B. Mukherjee** designed experiments, performed all the *in vivo* and majority of the *in vitro* experiments, data curation, formal analysis, methodology, writing original draft, review and editing; **A. Tiwari** performed the *in vitro* experiments given in Figure 1A, 1B, 1E, 1G and performed IHC of 46 samples (Figure 6). **A. Palo** performed the ChIP assay (Figure 5H); **N. Pattnaik** analysed and scored patient IHC samples, **S. Samantray** provided some of the patient samples, **M. Dixit** conceptualization, designed experiments, guided the research, formal analysis, supervision, funding acquisition, resources, writing-review and editing the manuscript.
