# Supplementary material for: Reduced expression of FRG1 facilitates breast cancer progression via GM-CSF/MEK-ERK axis by abating FRG1 mediated transcriptional repression of GM-CSF
Source: Cell Death Discov. 2022 Nov 3;8:442. doi: 10.1038/s41420-022-01240-w (PMC9633810; doi:10.1038/s41420-022-01240-w)
Supplement: Supplementary file 3 — Supplementary figures [file 41420_2022_1240_MOESM3_ESM.pdf]

Supplementary Figure. 1

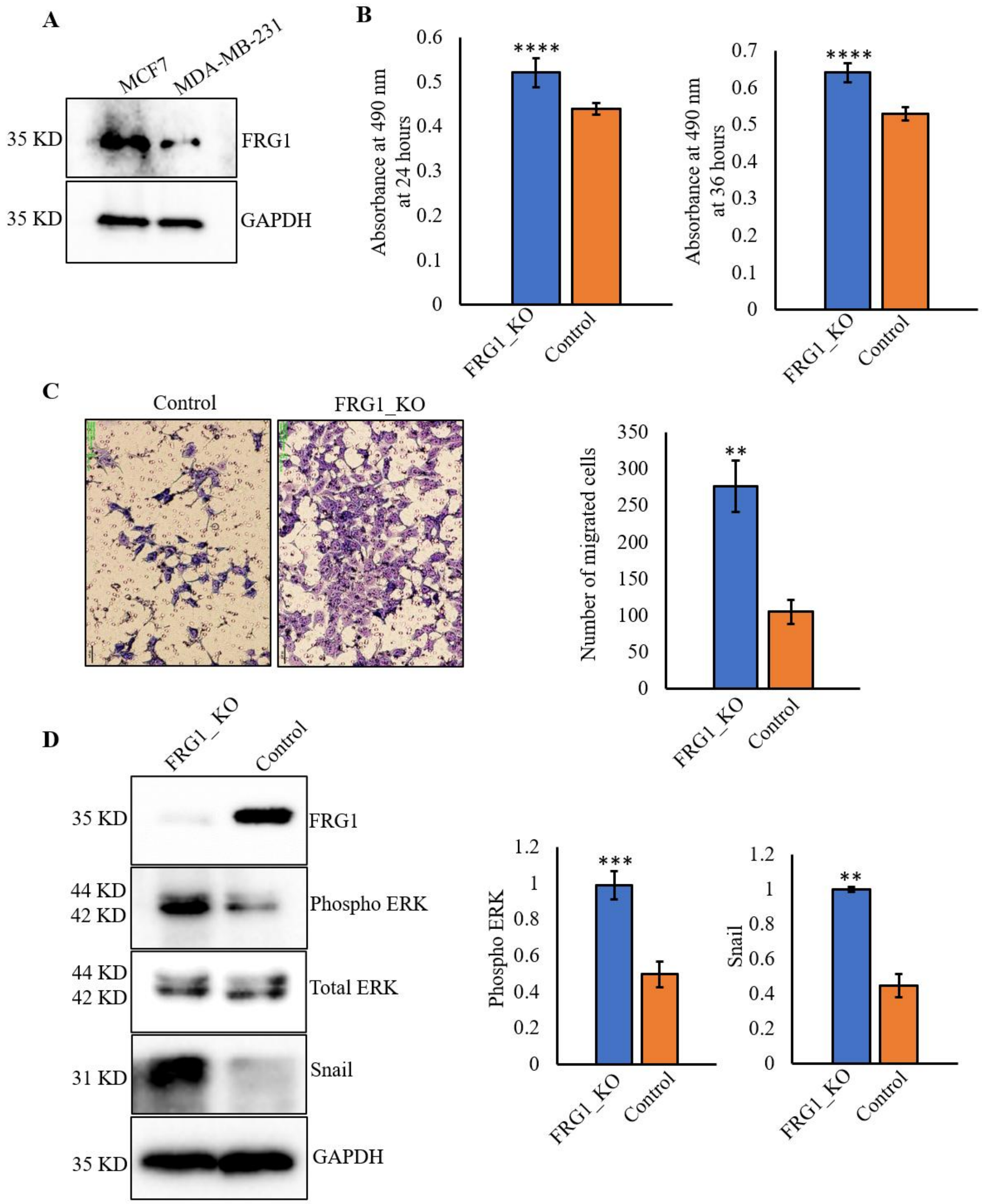

Supplementary Figure S1. Endogenous expression of FRG1 in breast cancer cell lines and effect of FRG1 knockout on tumorigenic properties. **A,** Western blot was done in two breast cancer cell lines of different molecular subtypes to observe the endogenous expression of FRG1. Representative Western blot image shows comparative expression of FRG1 in luminal (ER+) type breast cancer cell line MCF7 and TNBC cell line MDA-MB-231. **B-D,** Cell-based assays, and Western blot-based expression analysis were done in MCF7 cells with FRG1 knockout (FRG1_KO) and unmodified cells (control). **B,** Proliferative property of MCF7 cells in both the groups (FRG1_KO and control) was determined at 24 hours and 36 hours using MTS assay. Bar diagrams show OD values taken at 490 nm in FRG1_KO and Control groups. **C,** Representative images illustrate the migration of MCF7 cells in FRG1_KO and control groups. Scale bar, 50 µm. The bar diagram shows the number of migrated cells between the two groups. **D,** Representative immunoblots and corresponding bar graphs show the expression of pERK and EMT marker snail in FRG1_KO and control MCF7 cells. The intensity of the bands in each blot was measured by ImageJ software. Relative fold change was calculated in Excel by normalizing to loading control GAPDH. Two-tailed unpaired student's t test was used to compare the difference between the two groups. Shown results are representative of three independent experiments. Results are presented as mean ± SD. ns, $P > 0.05$, **, $P \leq 0.01$; ***, $P \leq 0.001$.

Supplementary Figure. 2

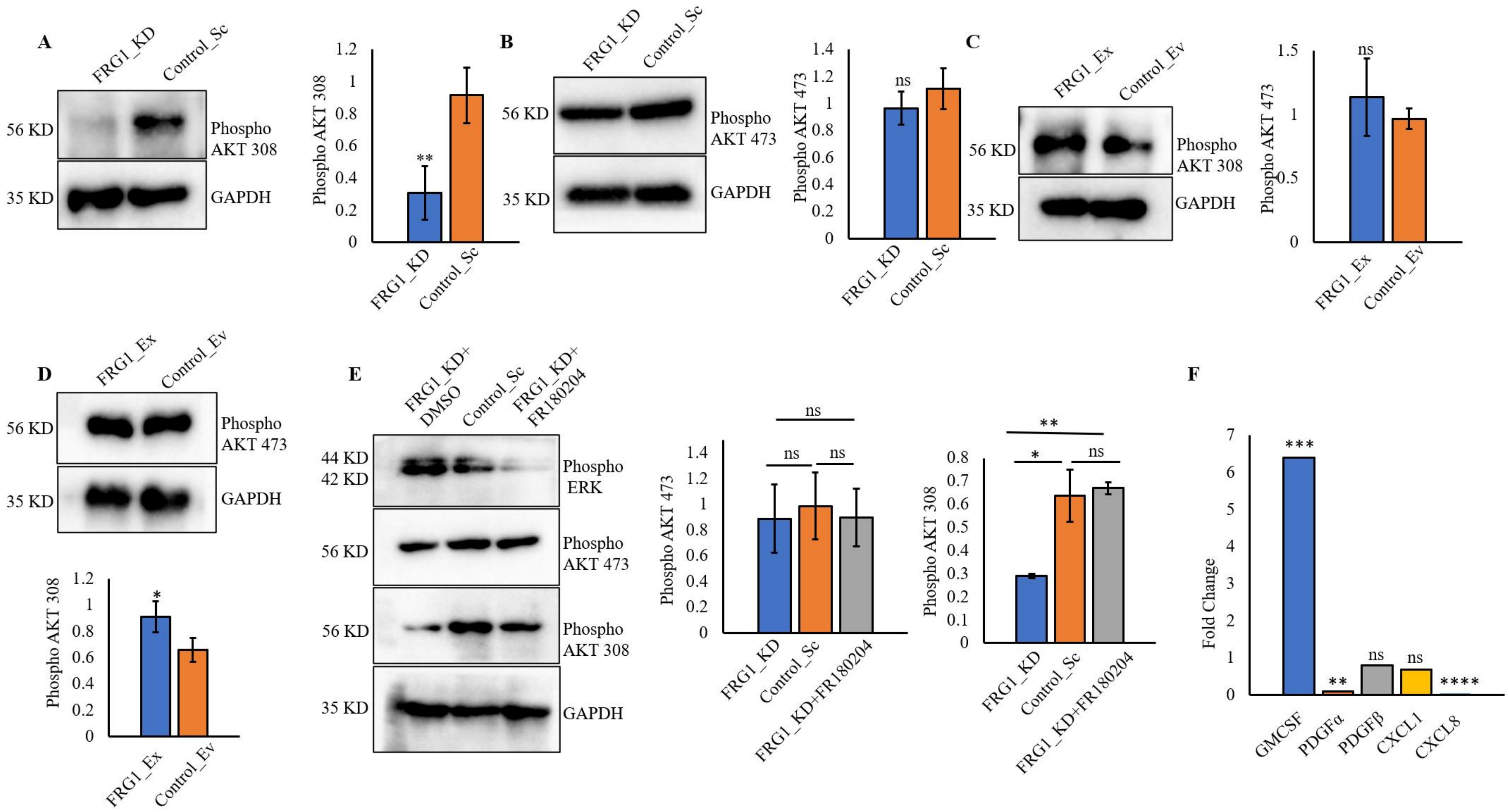

Supplementary Figure S2. Validation of effect of FRG1 on AKT activation and cytokine expression. In MCF7 cells, FRG1 levels were depleted (FRG1_KD) and elevated (FRG1_Ex); after that, they were subjected to immunoblotting to assess the effect of FRG1 modulation on AKT activation. **A-B,** Representative western blots and bar graphs showing the expression of pAKT 308 (A) and pAKT 473 (B) in FRG1 depleted MCF7 cell line (FRG1_KD) and its control (Control_Sc). **C-D,** Representative immunoblots, and bar graphs showing the expression of pAKT 308 (C) and pAKT 473 (D) due to elevated expression of FRG1 in MCF7 cells (FRG1_Ex) and its control (Control_Ev). **E,** MCF7, FRG1_KD cells were treated with 10μM of ERK inhibitor FR180204 (FRG1_KD+FR180204) and DMSO (FRG1_KD+DMSO), respectively, for 2 hours. The expression levels of pAKT 308/473 were measured by Western blot. Shown results are representative of three independent experiments. **F,** MCF7_FRG1_KD cells were treated with10μM of ERK inhibitor FR180204 (FRG1_KD+FR180204) for two hours. The bar graph shows the effect of ERK inhibition in the MCF7 cells with FRG1 reduction (FRG1_KD) on fold change in mRNA levels of GM-CSF, PDGFβ, PDGFα, CXCL1, and CXCL8 compared to Control_Sc as determined by qRT-PCR (n = 3). GAPDH has been used as an internal control. Results are shown as mean ± SD. Two-tailed unpaired students' t-test was used to compare the difference between the two groups. ns, P > 0.05, *, P ≤ 0.05, **, P ≤ 0.01; ***, P ≤ 0.001; ****, P ≤ 0.0001.

Supplementary Figure. 3

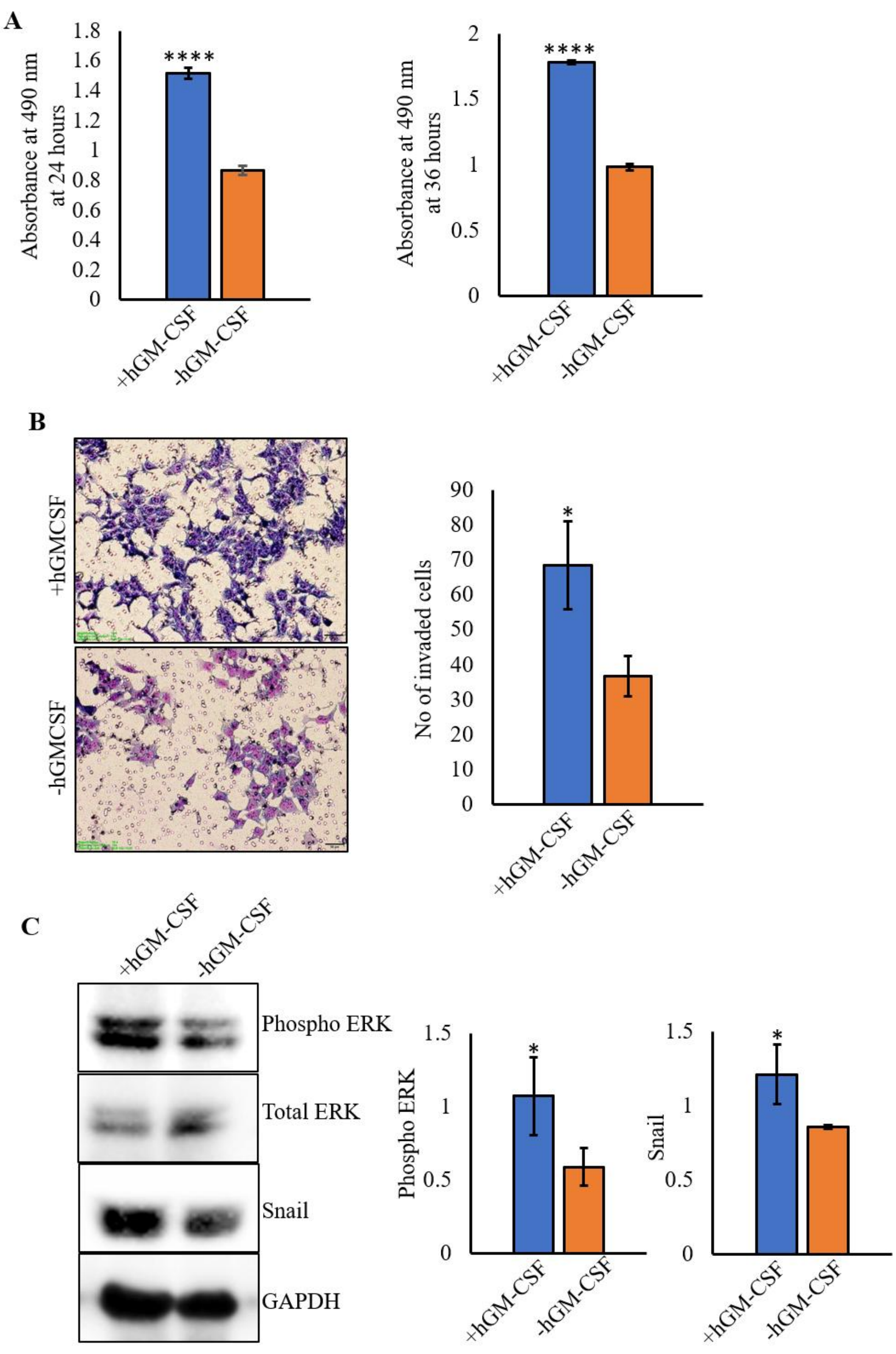

Supplementary Figure S3. Effect of GM-CSF on tumorigenic properties of MCF7 cell line. MCF7 cells were treated with 100 ng of human recombinant GM-CSF (+hGM-CSF) and PBS (-hGM-CSF), respectively, for an hour. After that effect of GM-CSF in MCF7 cells was assessed by MTS assay, invasion assay, and immunoblotting. **A,** The bar graph of the MTS assay shows the difference in cell proliferation in MCF7 cells treated with human recombinant GM-CSF (+hGM-CSF) and PBS (-hGM-CSF) at 24 and 36 hours. OD values were taken at 490 nm. **B,** Representative images and bar graph depict the invasion of MCF7 cells in +hGM-CSF and -hGM-CSF groups (n = 4). Scale bar, 50 µm. **C,** Representative Western blot images and densitometry-based bar graphs (n = 3) depict the effect of GM-CSF (+hGM-CSF vs. -hGM-CSF) on the levels of pERK and EMT marker snail. Two-tailed unpaired student's t-test was used to compare the difference between the two groups. *, $P \leq 0.05$; ****, $P \leq 0.0001$.

Supplementary Figure. 4

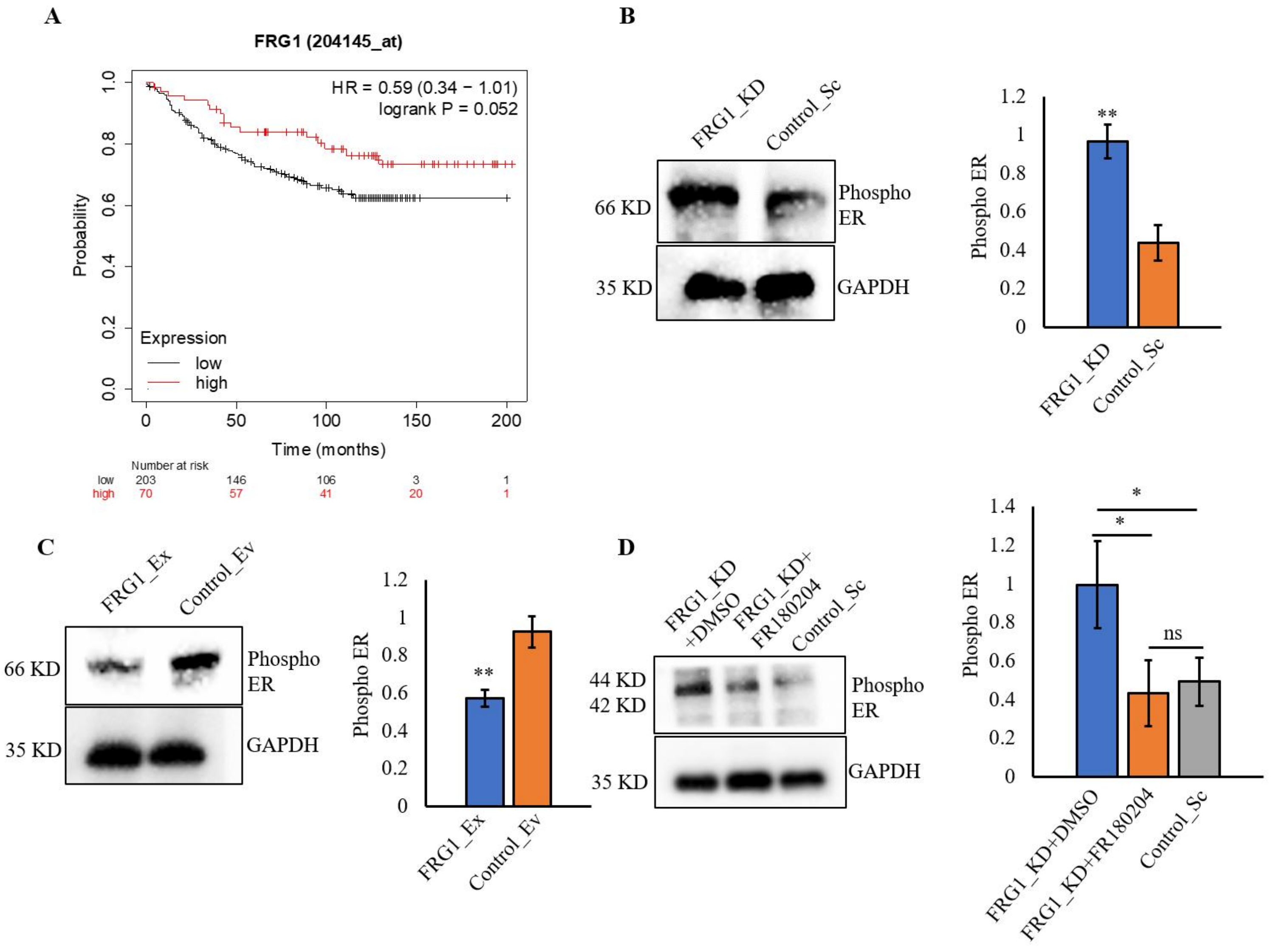

Supplementary Figure S4. Effect of FRG1 on breast cancer patient survival and the activation of ER. A, Analysis of combined datasets in Kaplan-Meir plotter shows the probability of recurrence-free survival in high FRG1 (n=70) and low FRG1 (n=203) groups containing wild-type p53. **B,** Western blots showing the expression of pER due to FRG1 knockdown in MCF7 cells (FRG1_KD) and Control_Sc. **C,** Immunoblots showing the effect of ectopic expression of FRG1 (FRG1_Ex) vs. Conrol_Ev on pER. **D,** MCF7_FRG1_KD cells were treated with 10 μM of ERK inhibitor FR180204 (FRG1_KD+FR180204) and DMSO (FRG1_KD+DMSO), respectively, for 2 hours. Levels of pER level have been measured by Western blot. Shown results are representative of three independent experiments. Two-tailed unpaired student's t-test was used to compare the difference between the two groups. Results are presented as mean ± SD. ns, $P > 0.05$, *, $P \leq 0.05$; **, $P \leq 0.01$.

Supplementary Figure. 5

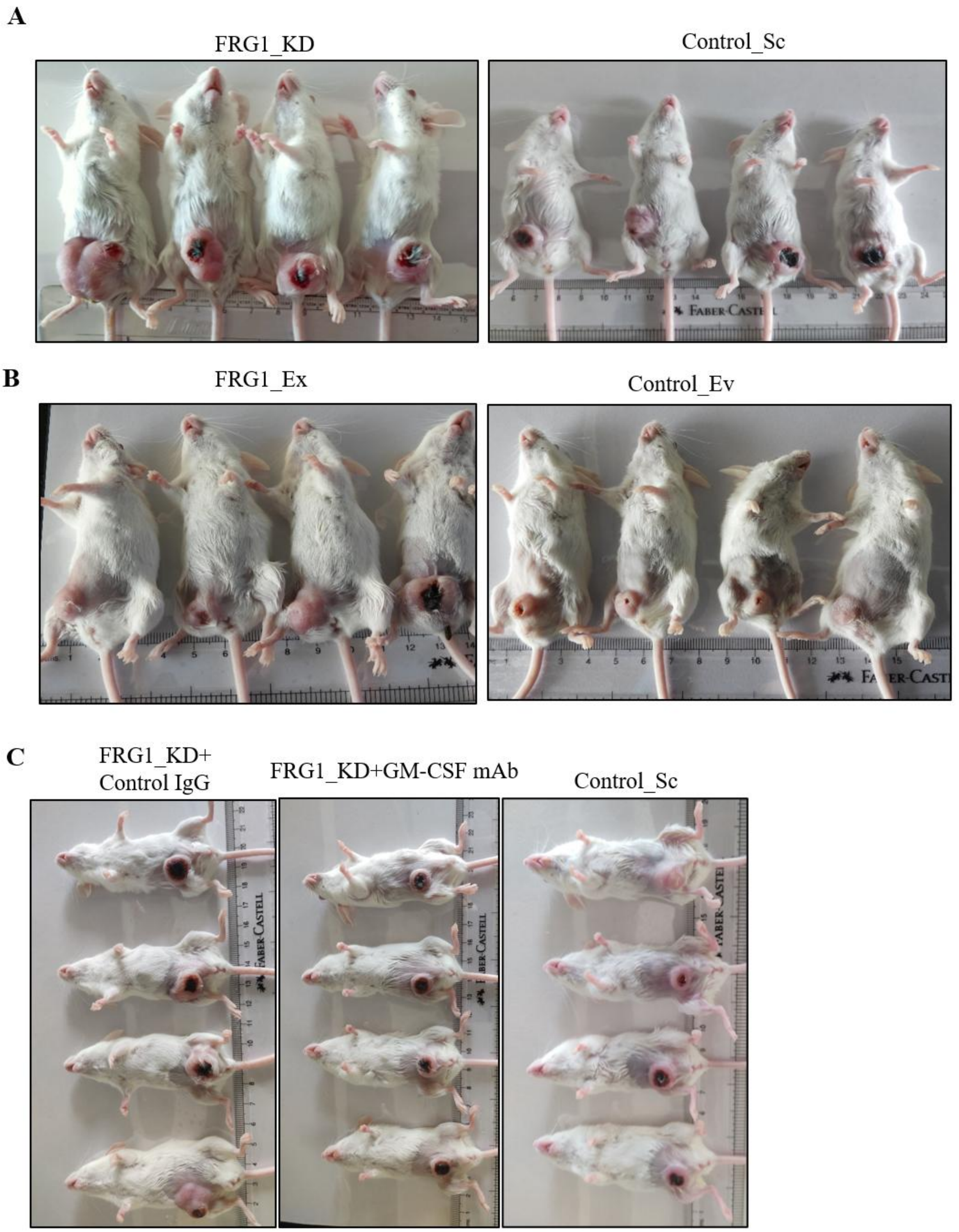

Supplementary Figure S5. Effect of anti-GM-CSF treatment on tumors derived from cell lines with altered FRG1 levels in BALB/c mice. **A-B,** Representative images showing the mice bearing the tumors, injected with 4T1 cells with (A) reduced (4T1_FRG1_KD) and (B) elevated levels of FRG1 (4T1_FRG1_Ex) along with their respective controls (n = 4). **C,** BALB/c mice were injected with 4T1_FRG1_KD cells to develop tumors along with the corresponding control (Control_Sc). After 7 days 4T1_FRG1_KD group mice was treated with anti GM-CSF antibody (n = 4) or control_IgG (n = 4), till day 21. Images of tumor-bearing mice from the three groups (FRG1_KD+Control IgG, FRG1_KD+GM-CSF mAb, and Control_Sc).
