# Supplementary material for: Reduced expression of FRG1 facilitates breast cancer progression via GM-CSF/MEK-ERK axis by abating FRG1 mediated transcriptional repression of GM-CSF
Source: Cell Death Discov. 2022 Nov 3;8:442. doi: 10.1038/s41420-022-01240-w (PMC9633810; doi:10.1038/s41420-022-01240-w)
Supplement: Supplementary file 4 — Supplementary Materials and methods [file 41420_2022_1240_MOESM4_ESM.docx]

**Supplementary Information**

**Supplementary materials and methods**

**Cell culture, plasmids and generation of stable cell lines**

Human breast cancer cell lines MCF7 and MDA-MB-231 were purchased from National Center for Cell Science (NCCS, Pune, India) cell repository. The repository itself authenticated cell lines with 16 STR markers (AmpFISTR Identifier Plus PCR Amplification Kit, Applied BioSystems) and checked for mycoplasma contamination. Experiments were started as soon as the procurement was done. MCF7 cells were maintained in DMEM (Himedia, Mumbai, India) supplemented with 10% FBS (Himedia, US origin) and 1X Penicillium-Streptomycin-Amphotericin B (PSA; Himedia, Mumbai, India). MDA-MB-231 cells were cultured in RPMI (Himedia, Mumbai, India) containing 15% FBS (US origin, Himedia) and 1X PSA. Mouse mammary carcinoma cell line 4T1 was obtained from American Type Culture Collection (ATCC, MNZ, USA) and maintained in RPMI with 10% FBS and 1X PSA. Cells were grown at 37ºC temperature and 5% CO2. FRG1 knockdown (pLKO.1_FRG1sh, TRCN0000075012) and expression vectors (HsCD004 21091 PLX304_FRG1) were purchased from Sigma and Harvard repository, respectively. FRG1 knockdown and expression vectors were transfected into the cells using Lipofectamine 3000 (Invitrogen) as per the manufacture’s protocol. Single cell-derived colonies were selected in puromycin (2µg/ml) (Sigma Aldrich, MO, USA) and blasticidin (10µg/ml) (Sigma Aldrich, MO, USA) for knockdown and overexpression vector, respectively. Stable lines were confirmed by Western blot.

**Generation of FRG1 knockout cell line**

Single-guide RNA (sgRNA) was designed in the CHOP-CHOP version 3 web tool (<https://chopchop.cbu.uib.no/>) and subsequently cloned into the plasmid pSpCas9(BB)-2A-Puro (PX459) V2.0 (Addgene, MA, USA) with the BbsI site at the end of the human U6 promoter. sgRNA primers that were used to knockout the FRG1 gene contain the sequence 5’- TTCTGGACGAGTATGTGAGT-3’ and 5’-AAGACCTGCTCATACACTCA-3’. After transfecting MCF7 cells with the vector, single cell-derived colonies were selected using puromycin (5µg/ml) (Sigma). Knockout was confirmed by Western blot.

**Matrigel Invasion Assay**

To perform the invasion assay, cells were initially grown for 12 hours in the reduced serum medium (2% FBS). Next, 0.5×10^6^ cells were plated onto the membrane filter inserts (8µm pore size, Merck, MA, USA) coated with growth factor reduced matrigel (1mg/ml) (Corning, NY, USA), in 500 µl of serum-free medium. Inserts were placed in a 12-well plate where the lower chambers were filled with 1 ml of complete medium and kept at 37°C in a humidified chamber containing 5% CO2. After 24 hours, inserts were taken out, and cells were fixed with methanol (Himedia, Mumbai, India), and stained with Giemsa (Himedia). On the upper side of the matrigel, Uninvaded cells were removed with a cotton bud. Images were captured at X10 magnification in an upright brightfield microscope (Olympus, Tokyo, Japan).

**MTS assay for cell proliferation**

For proliferation assay, 5000 cells were plated into a 96-well plate in the complete medium. After the specified time period, 20 µl of CellTiter 96® AQueous One Solution Reagent (Promega, WI, USA) was added to each well and incubated at 37°C for an hour. After that, absorbance was recorded at 490 nm using a Varioscan multimode microplate reader (Thermo Scientific).

**Migration Assay**

We plated 1×10^6^ cells per well in a 6-well plate. At 90% confluency, the complete cell culture medium was replaced by a reduced serum medium, and a scratch was made using a 200 µl pipette tip through the middle of the plate. Subsequently, each well was washed twice with 1X PBS to get rid of cellular debris. Images of the wounds were taken at 0, 24 and 36 hours in an inverted microscope (Nikon, Tokyo, Japan) using X10 magnification. The gap closure was quantified by ImageJ software (NIH, MD, USA).

**Colony formation assay**

Seven hundred cells were plated into a 6-well plate and kept in the incubator at 37°C in 5% CO2. After 15 days, cells were washed with 1x PBS and fixed using methanol for 10 minutes. Next, the cells were stained with Giemsa (Himedia) for 15 minutes, followed by PBS wash twice. Cell colonies were photographed by using a digital camera (Nikon). Colony number was counted in ImageJ software (NIH).

**Tumor development assays**

Female BALB/c mice were used to carry out all the animal experiments. To satisfy the inclusion criteria, healthy mice of same age group (6-8 weeks) were included in the study. Instead of blinding, the animals were assigned to treatment and control group randomly. For tumor development, 2 X 10^6^ 4T1 cells with altered FRG1 expression and respective control were orthotopically injected into the mammary fat pad of mice (n = 4 in each group). Tumor size was measured with a digital Vernier caliper, on every third days, and the volume was calculated using the formula (L X W^2^)/2, (L = length, W = width). After 30 days, mice were sacrificed, and tumors were excised, weighed, and photographed. To examine the spontaneous metastasis, 1X10^5^ 4T1_FRG1_KD cells were injected into the tail vein of the mice (n = 4) along with the control group (n = 4). After 14 days, mice were sacrificed, and their lungs were excised out to count the metastatic nodules.

To investigate the effect of GM-CSF inhibition in vivo, tumor was developed in BALB/c mice using 4T1_FRG1_KD cells (n = 4). Once the palpable tumor was present (day 7), anti-mouse GM-CSF neutralizing antibody (10 mg/kg body weight) (#MP1-22E9, Bio-Xcell, NH, USA) and control IgG (10 mg/kg body weight) were administrated intraperitoneally every alternate day till day 21. After that, the mice were euthanized, and tumors were excised and stored in PBS. Animal and tumor images were recorded using a digital camera (Nikon). The tumors were minced, and protein lysate was prepared for Western blotting as mentioned in the earlier section.

**Pharmacological compounds used for inhibition and activation assays**

For ERK inhibition, cells were treated with 10 µM of ERK inhibitor (#559388, Merck, MA, USA) for 2 hours to perform the downstream experiments. For ERK activation, we used 5µM of ERK activator Ceramide (#SC-3527, Santa-Cruz, CA, USA) for 2 hours. We inhibited CXCR2 receptor of the cells by administrating CXCR2 antagonist Cpd 19 (#239819, Merck, MA, USA) for 2 hours. For *in vitro* GM-CSF inhibition and activation experiments, cells were treated with 10 µg/ml of anti-human GM-CSF antibody (#502206, BioLegend, CA, USA) and 100 ng/ml of human recombinant GM-CSF (#G5035, Sigma Aldrich, MO, USA) for 30 minutes and 1 hour, respectively. All the inhibitions and activations were initially standardized by treating the cells with different concentrations of the compounds at different time points. To achieve the minimum side effects due to the compounds, the least concentration was used to perform the experiments.
