# Supplementary material for: Reduced expression of FRG1 facilitates breast cancer progression via GM-CSF/MEK-ERK axis by abating FRG1 mediated transcriptional repression of GM-CSF
Source: Cell Death Discov. 2022 Nov 3;8:442. doi: 10.1038/s41420-022-01240-w (PMC9633810; doi:10.1038/s41420-022-01240-w)
Supplement: Supplementary file 5 — Supplementary Table 1 [file 41420_2022_1240_MOESM5_ESM.docx]

**Supplementary Table 1:** List of qRT-PCR primers

| **S. No.** | **Primer Name** | **Primer sequences (5' - 3')** |
| --- | --- | --- |
| 1 | FRG1 F | TGATATTGTTGGAATCTGGTGGACA |
| 2 | FRG1 R | CCATTGTCGAGTGCATGTATATAGG |
| 3 | GAPDH F | ACCCAGAAGACTGTGGATGG |
| 4 | GAPDH R | TCTAGACGGCAGGTCAGGTC |
| 5 | GM-CSF F | CTGGAGCTGTACAAGCAGGG |
| 6 | GM-CSF R | ACAGGAAGTTTCCGGGGTTG |
| 7 | PDGFA F | GCCAACCAGATGTGAGGTGA |
| 8 | PDGFA R | GGAGGAGAACAAAGACCGCA |
| 9 | PDGFB F | ACCTGCGTCTGGTCAGC |
| 10 | PDGFB R | ATCTTCCTCTCCGGGGTCTC |
| 11 | CXCL1 F | AACCGAAGTCATAGCCACAC |
| 12 | CXCL1 R | GTTGGATTTGTCACTGTTCAGC |
| 13 | CXCL8 F | ACCGGAAGGAACCATCTCAC |
| 14 | CXCL8 R | GGCAAAACTGCACCTTCACAC |
| 15 | CSF2 (ChIP)F | GAGGGGCACAGTTTGGACTT |
| 16 | CSF2 (ChIP)R | CAAAGGCCCCTGGGATTACA |
