# Supplementary material for: Reduced expression of FRG1 facilitates breast cancer progression via GM-CSF/MEK-ERK axis by abating FRG1 mediated transcriptional repression of GM-CSF
Source: Cell Death Discov. 2022 Nov 3;8:442. doi: 10.1038/s41420-022-01240-w (PMC9633810; doi:10.1038/s41420-022-01240-w)
Supplement: Supplementary file 6 — Supplementary Table 2 [file 41420_2022_1240_MOESM6_ESM.docx]

**Supplementary Table 2:** List of primary antibodies used in Western blots

| **Sr. No.** | **Name of antibody** | **Dilution** | **Brand** | **Catalogue no** |
| --- | --- | --- | --- | --- |
| 1 | Anti- FRG1 | 1:10000 | Abcam, MA, USA | ab181083 |
| 2 | Anti-phospho-ERK | 1:1000 | CST, MA, USA | 4370S |
| 3 | Anti-ERK | 1:1000 | CST, MA, USA | 9102S |
| 4 | Anti-phospho-MEK | 1:1000 | CST, MA, USA | 9154S |
| 5 | Anti-MEK | 1:1000 | CST, MA, USA | 9122S |
| 6 | Anti-Snail | 1:1000 | CST, MA, USA | 3879S |
| 7 | Anti-Slug | 1:1000 | CST, MA, USA | 9585S |
| 8 | Anti-Twist | 1:1000 | CST, MA, USA | 46702S |
| 9 | Anti-phospho-AKT 308 | 1:1000 | CST, MA, USA | 13038S |
| 10 | Anti-phospho-AKT 473 | 1:1000 | CST, MA, USA | 4060S |
| 11 | Anti-AKT | 1:1000 | CST, MA, USA | 4691S |
| 12 | Anti-phospho p53 | 1:1000 | CST, MA, USA |  |
| 13 | Anti-phospho ER | 1:1000 | CST, MA, USA | 2511S |
| 14 | Anti-phospho p38 | 1:1000 | CST, MA, USA | 9211S |
| 15 | Anti-GAPDH | 1:10000 | Abgenex, India | 10-10011 |
