# Supplementary material for: Reduced expression of FRG1 facilitates breast cancer progression via GM-CSF/MEK-ERK axis by abating FRG1 mediated transcriptional repression of GM-CSF
Source: Cell Death Discov. 2022 Nov 3;8:442. doi: 10.1038/s41420-022-01240-w (PMC9633810; doi:10.1038/s41420-022-01240-w)
Supplement: Supplementary file 7 — Original Data File [file 41420_2022_1240_MOESM7_ESM.pdf]

ORIGINAL UNCROPPED FIGURE 2

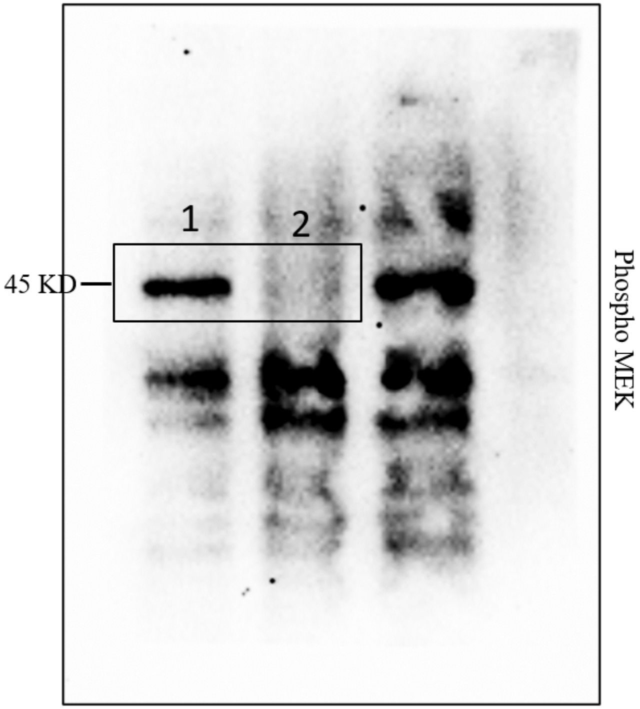

Fig. 2A (Lane 1,2 are on final figure)

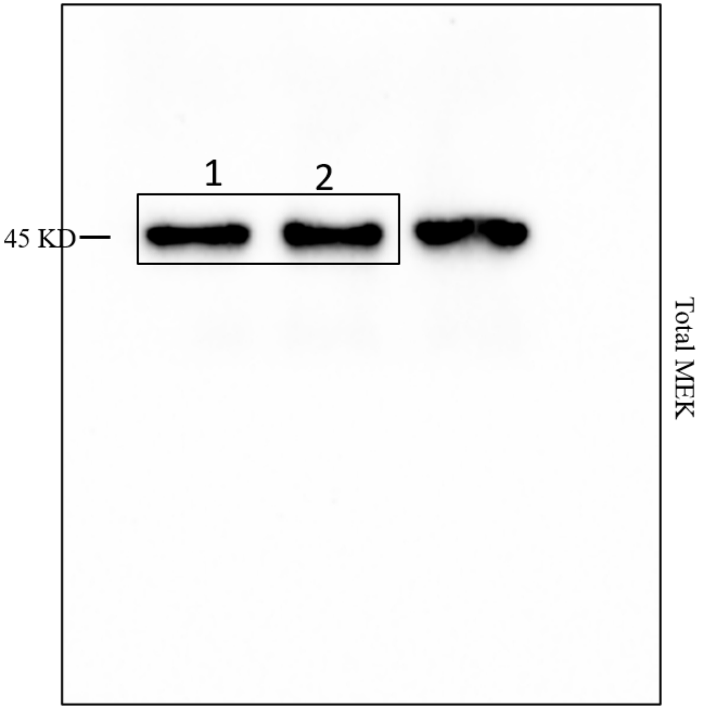

Fig. 2A (Lane 1,2 are on final figure)

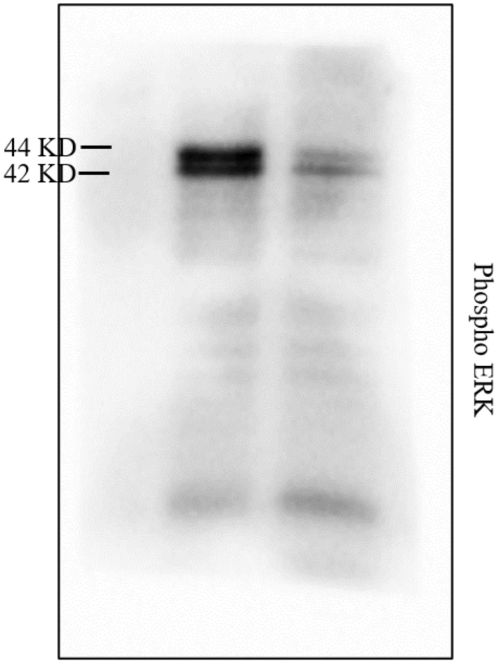

Fig. 2A

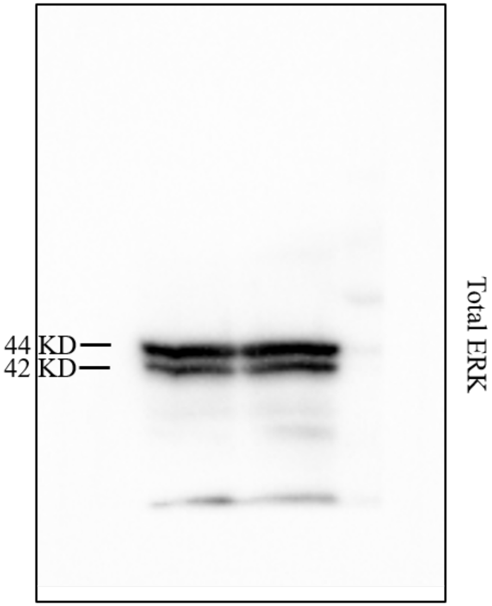

Fig. 2A

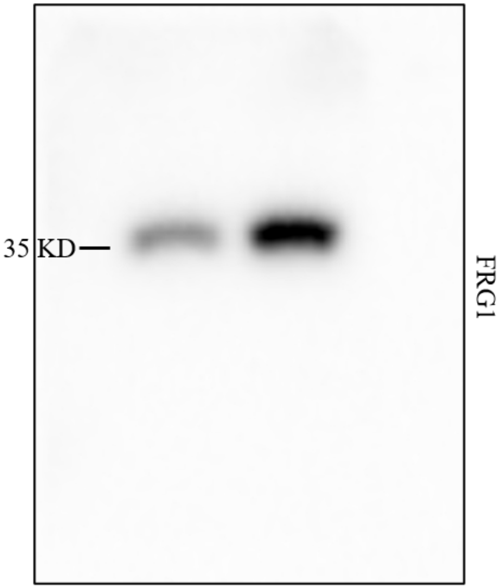

Fig. 2A

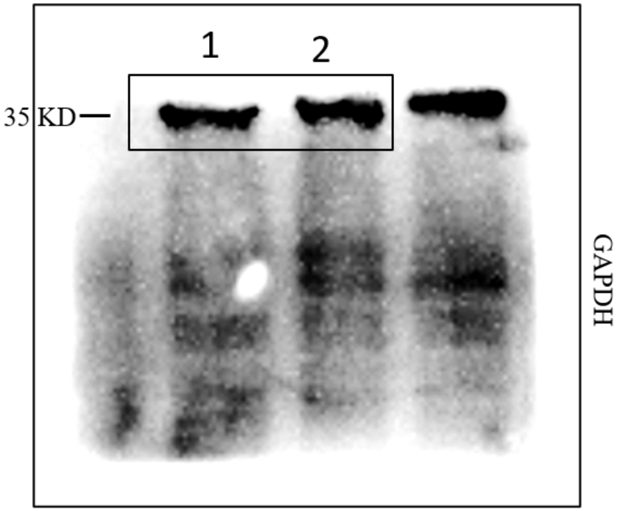

Fig. 2A (Lane 1,2 are on final figure)

ORIGINAL UNCROPPED FIGURE 2

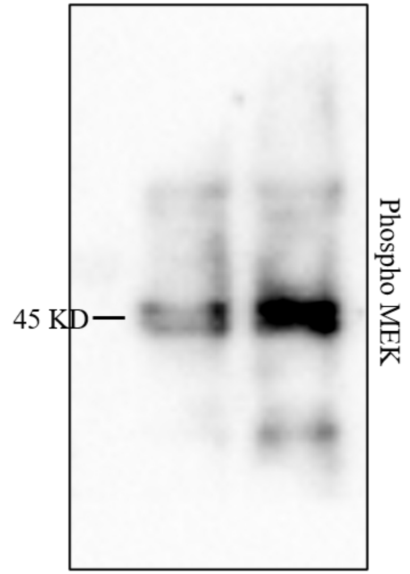

Fig. 2B

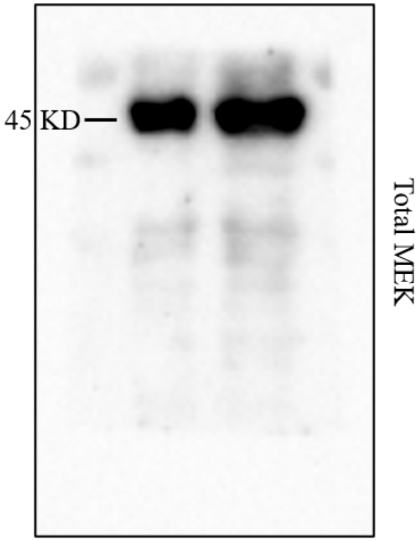

Fig. 2B

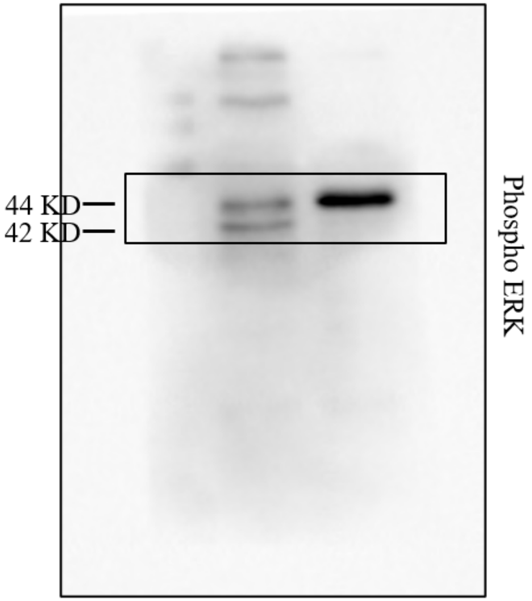

Fig. 2B

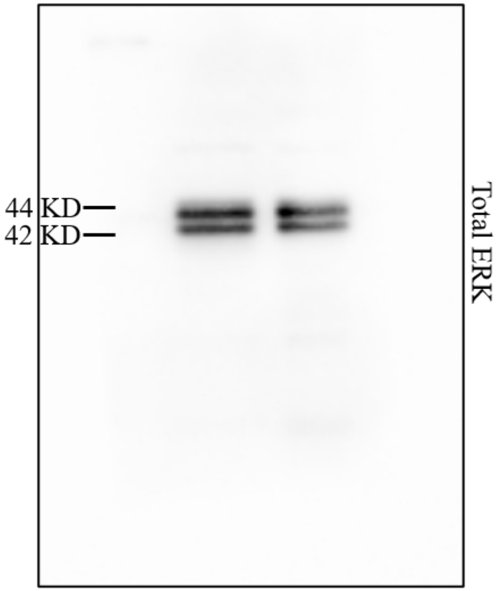

Fig. 2B

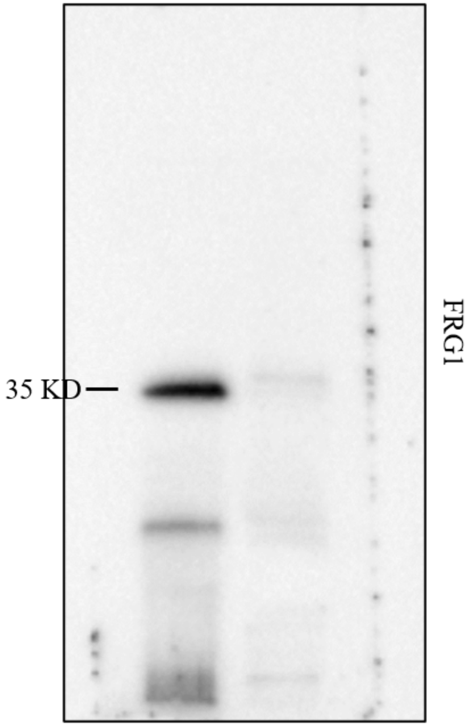

Fig. 2B

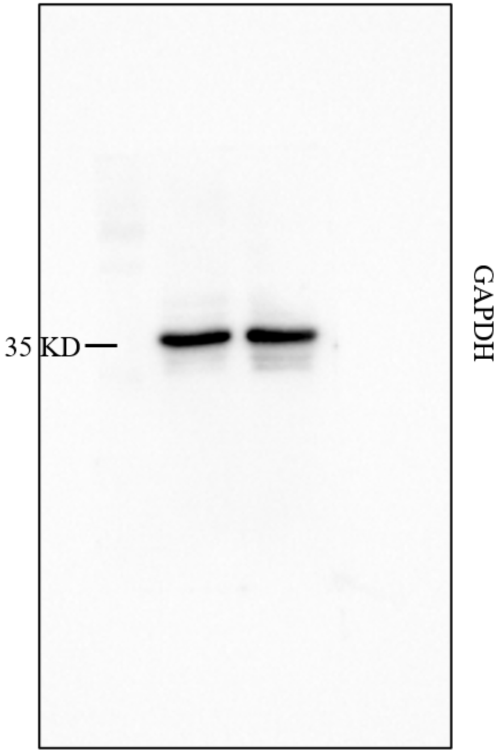

Fig. 2B

ORIGINAL UNCROPPED FIGURE 2

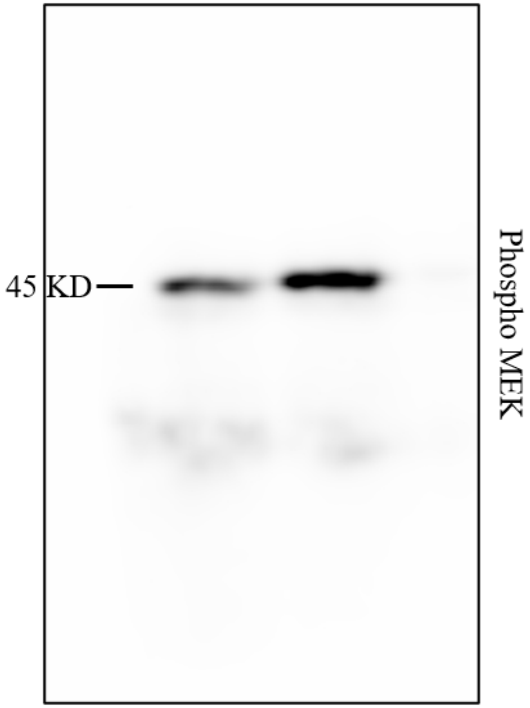

Fig. 2C

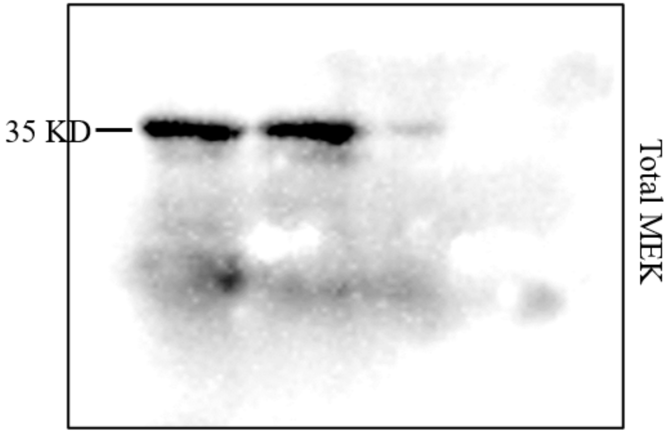

Fig. 2C

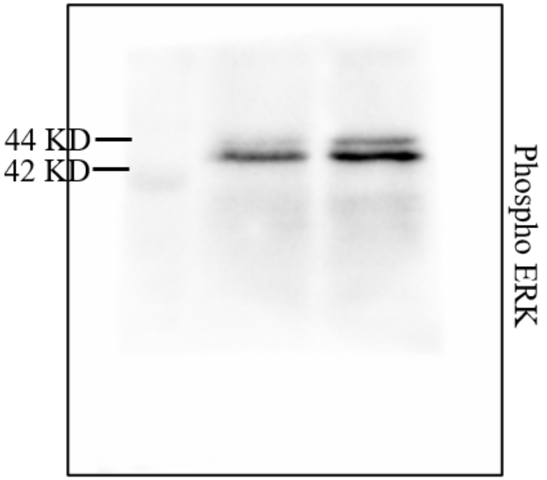

Fig. 2C

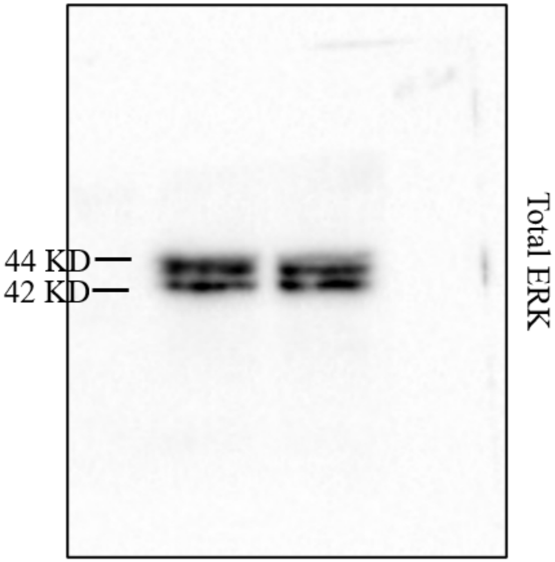

Fig. 2C

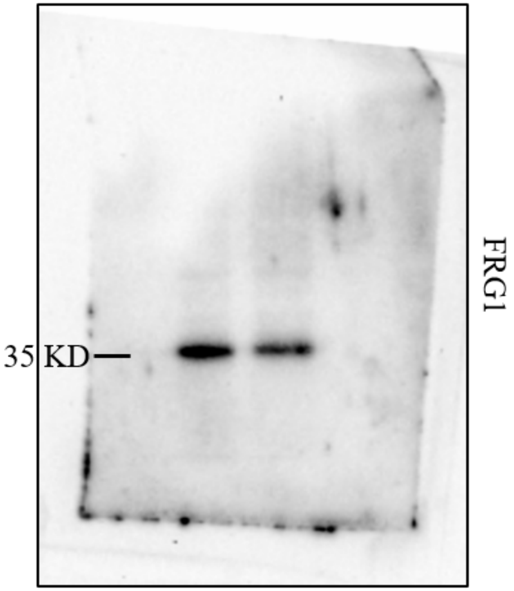

Fig. 2C

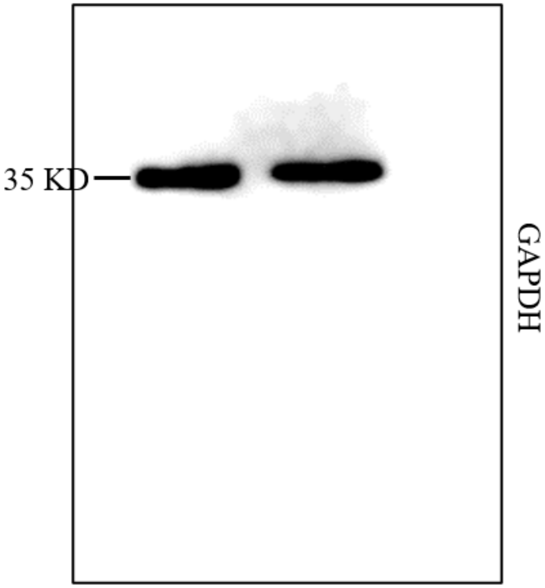

Fig. 2C

ORIGINAL UNCROPPED FIGURE 2

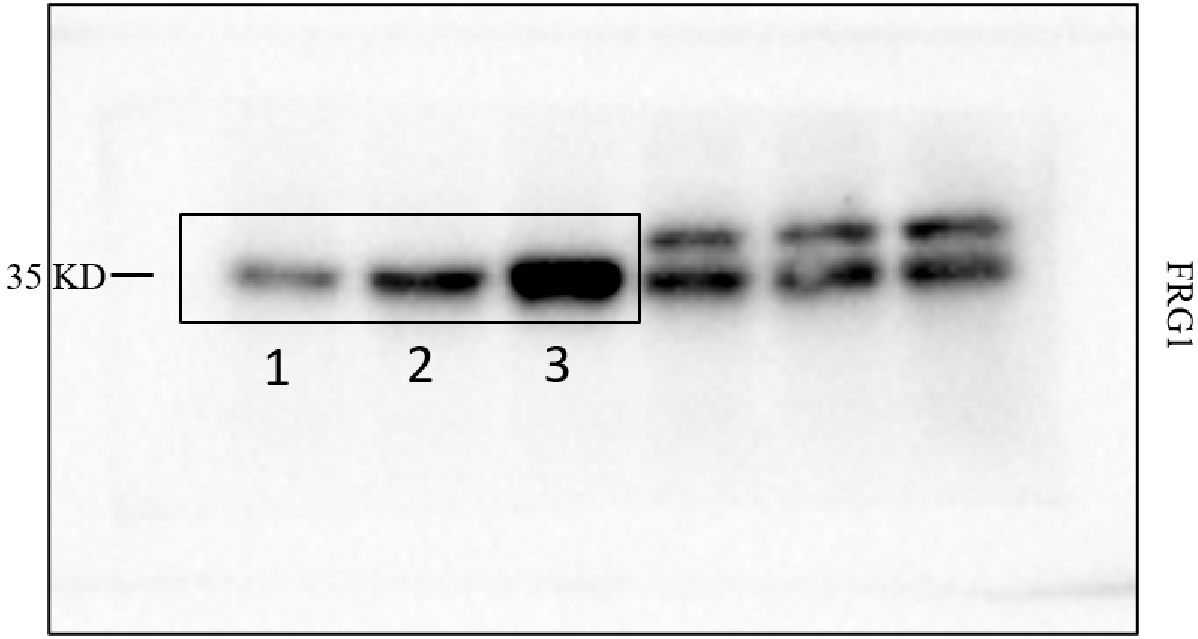

Fig. 2D (Lane 1,2,3 are on final figure)

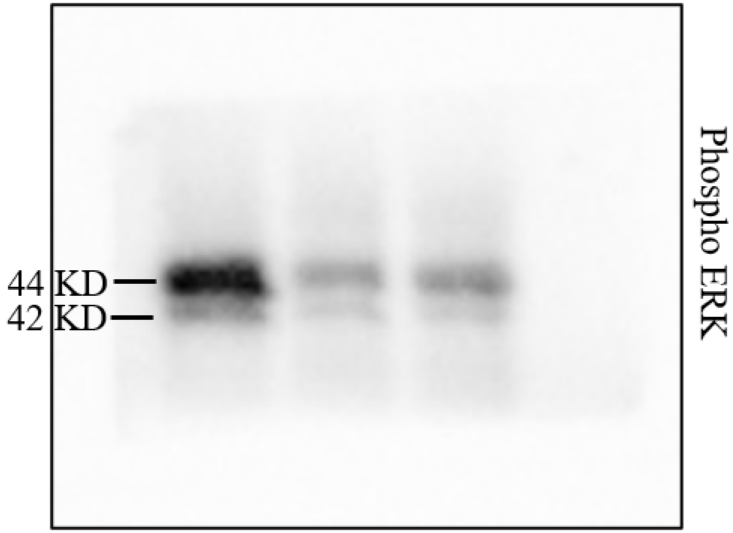

Fig. 2D

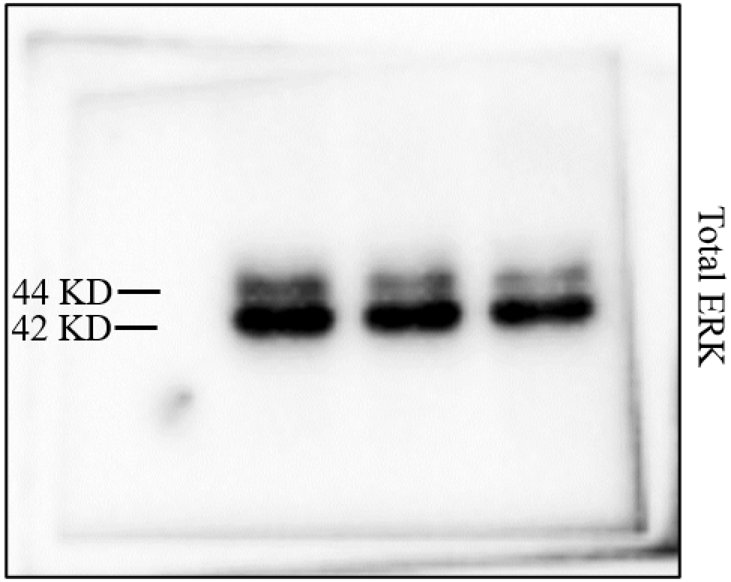

Fig. 2D

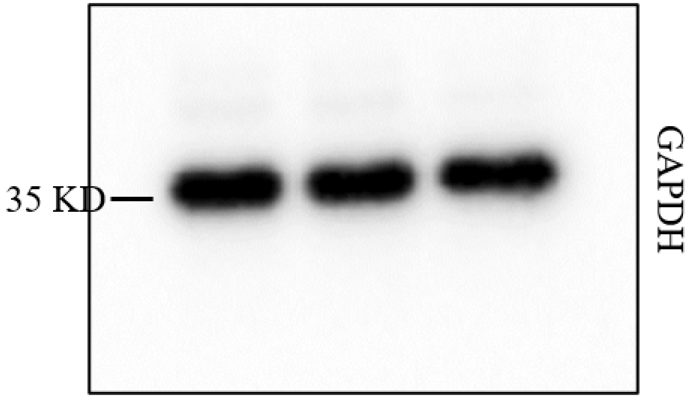

Fig. 2D

ORIGINAL UNCROPPED FIGURE 3

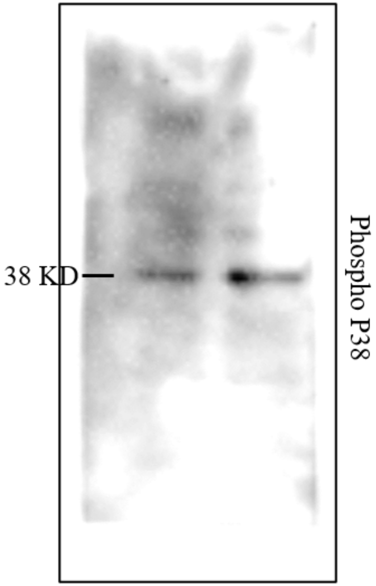

Fig. 3C

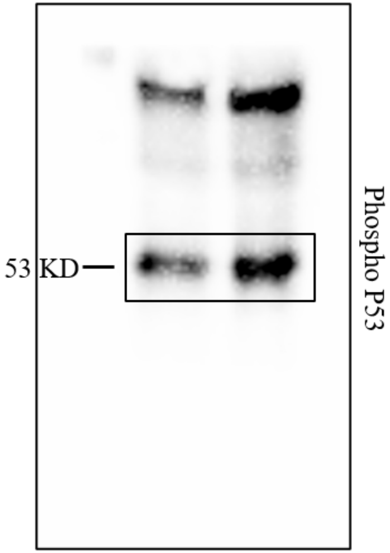

Fig. 3C

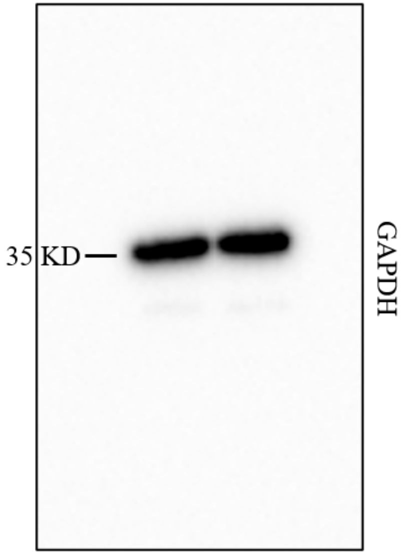

Fig. 3C

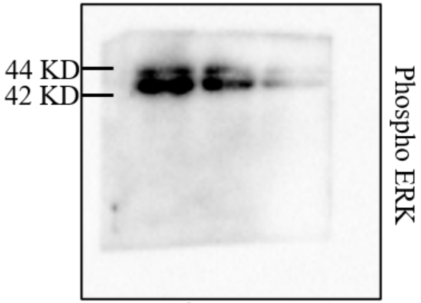

Fig. 3D

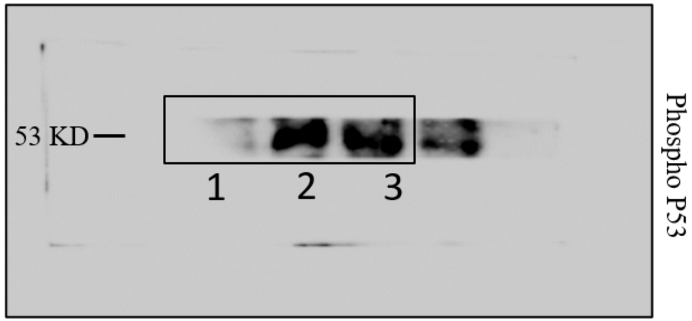

Fig. 3D

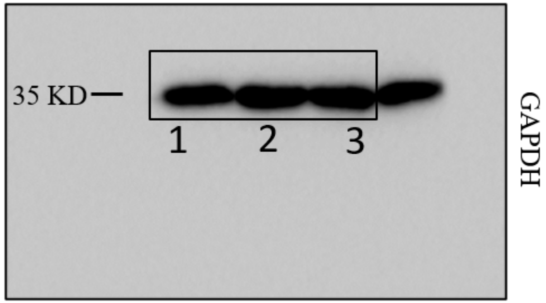

Fig. 3D (Lane 1,2,3 are on final figure)

ORIGINAL UNCROPPED FIGURE 4

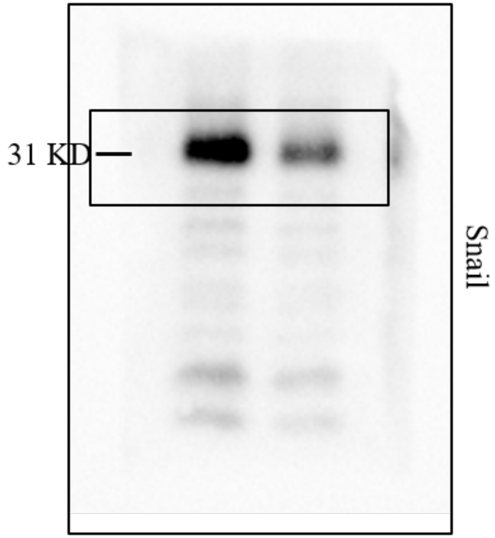

Fig. 4A

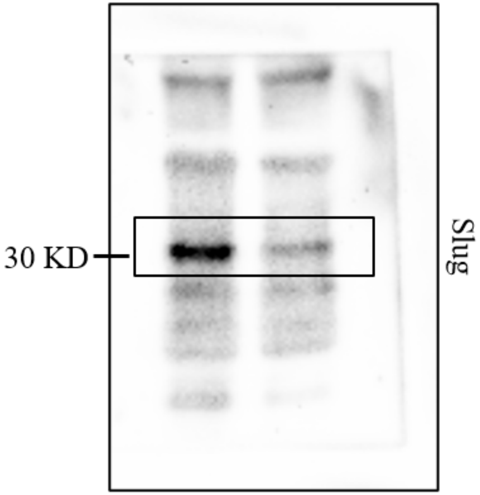

Fig. 4A

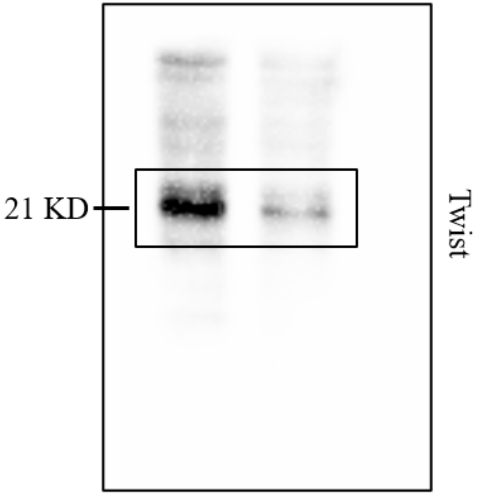

Fig. 4A

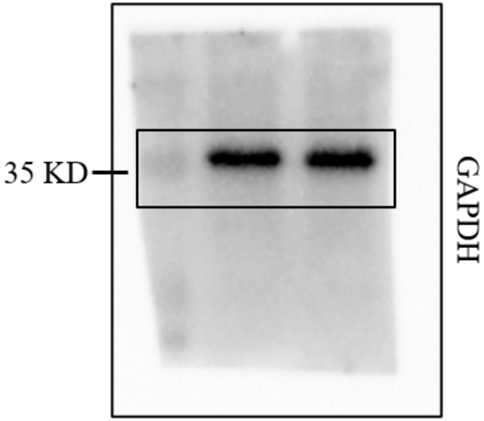

Fig. 4A

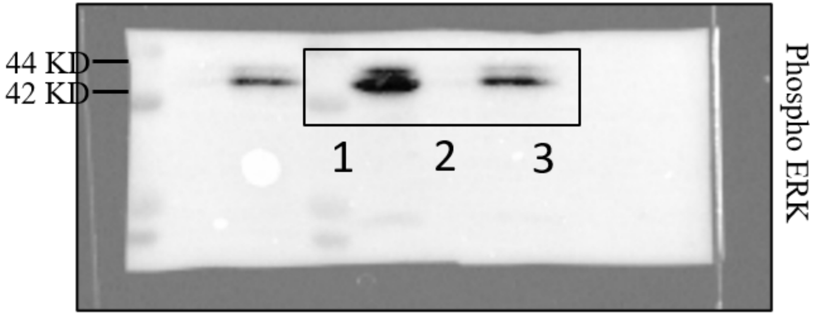

Fig. 4B (Lane 1,2,3 are on final figure)

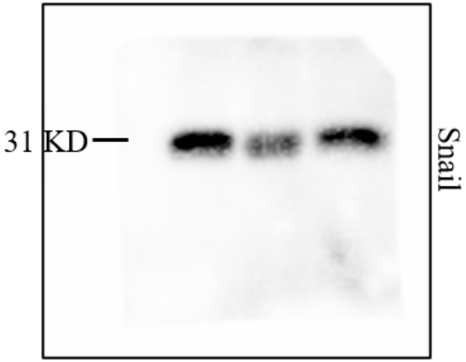

Fig. 4B

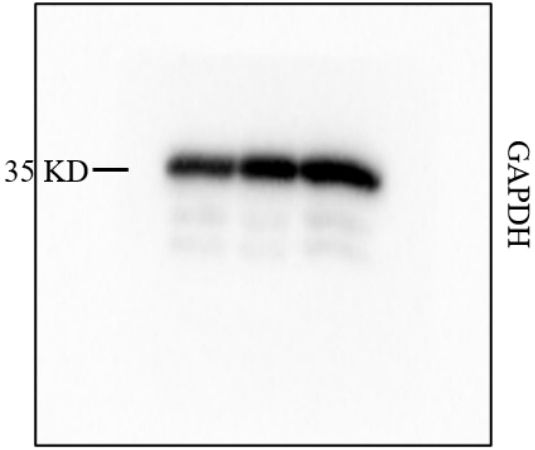

Fig. 4B

ORIGINAL UNCROPPED FIGURE 4

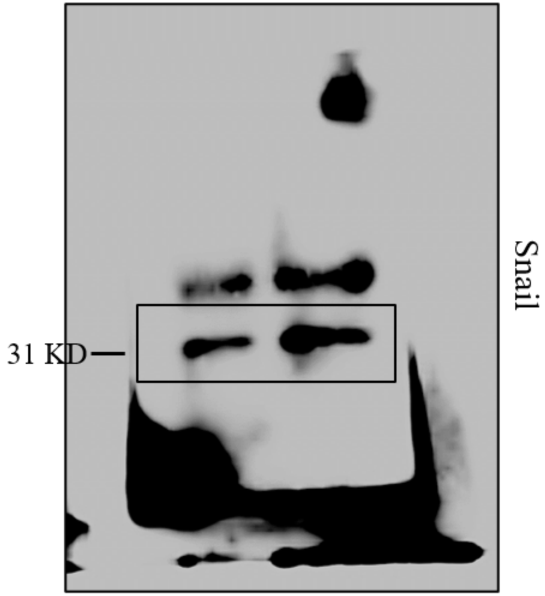

Fig. 4D

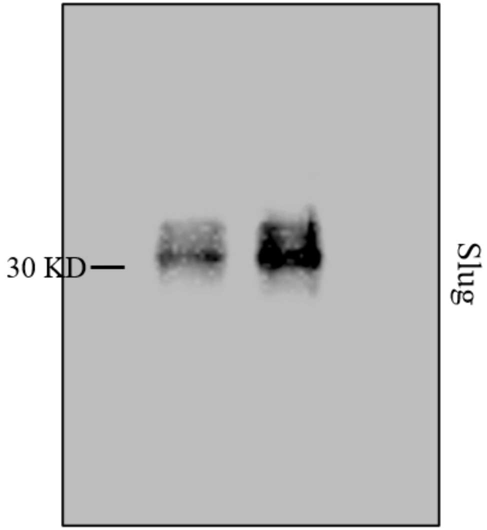

Fig. 4D

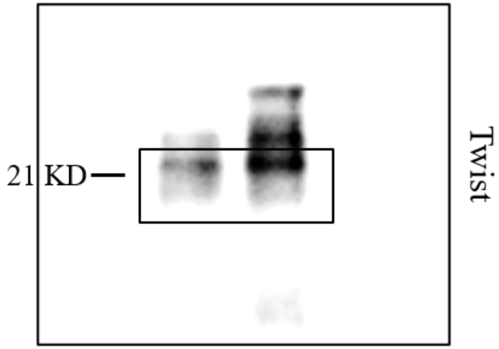

Fig. 4D

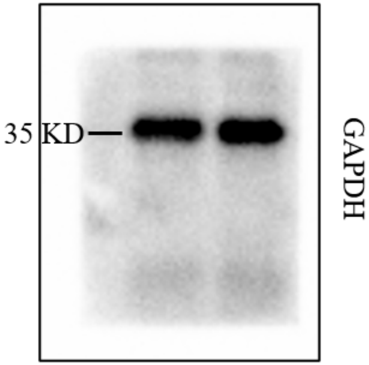

Fig. 4D

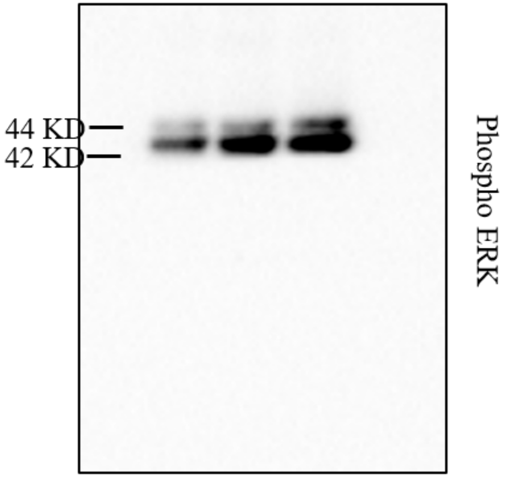

Fig. 4E

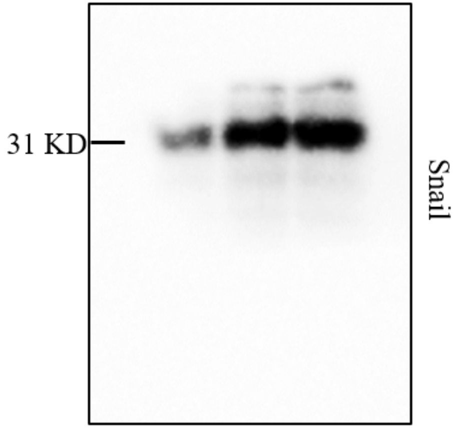

Fig. 4E

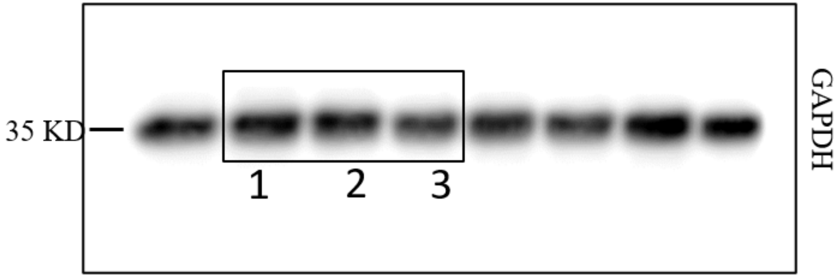

Fig. 4E (Lane 1,2,3 are on final figure)

ORIGINAL UNCROPPED FIGURE 4

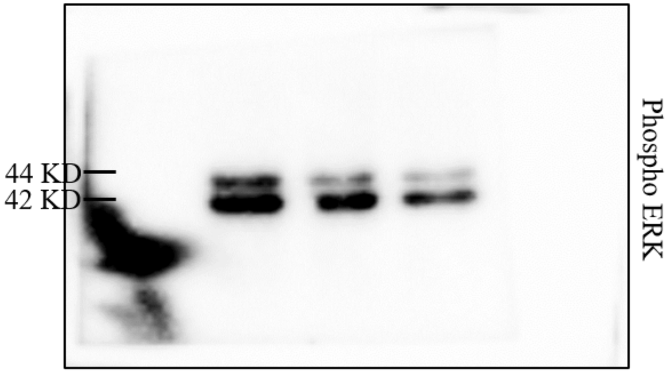

Fig. 4I

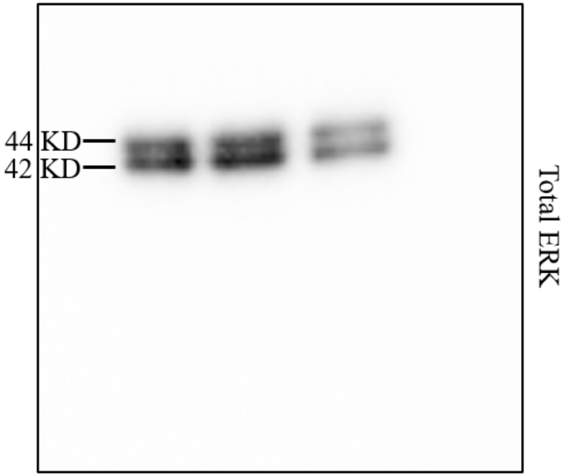

Fig. 4I

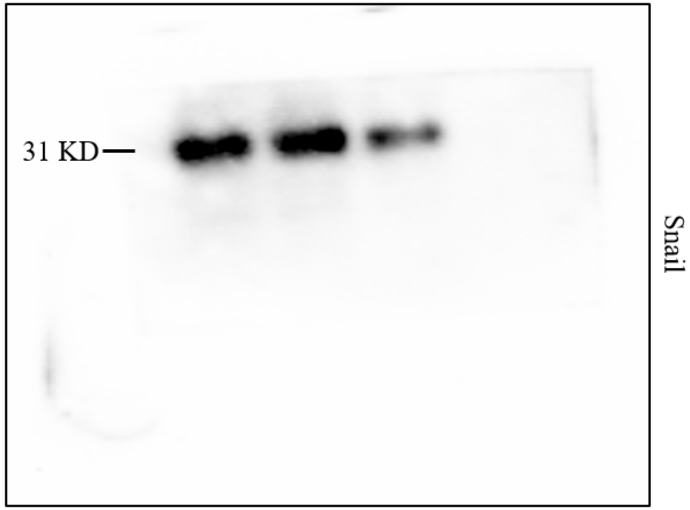

Fig. 4I

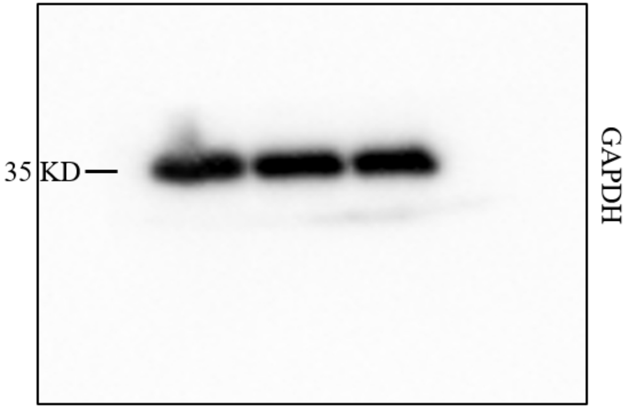

Fig. 4I

ORIGINAL UNCROPPED FIGURE 5

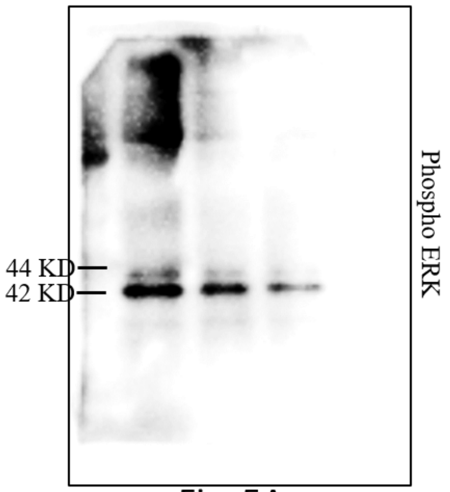

Fig. 5A

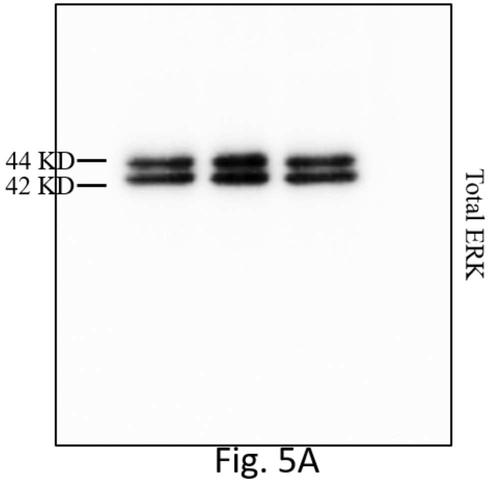

Fig. 5A

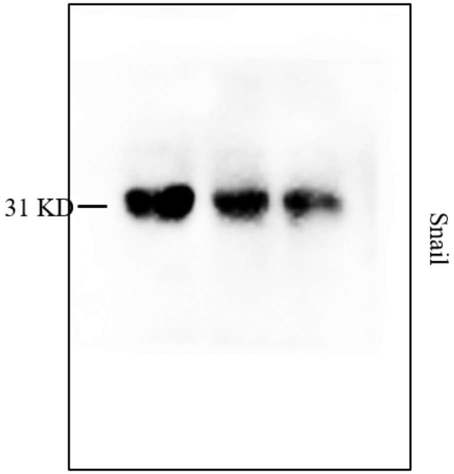

Fig. 5A

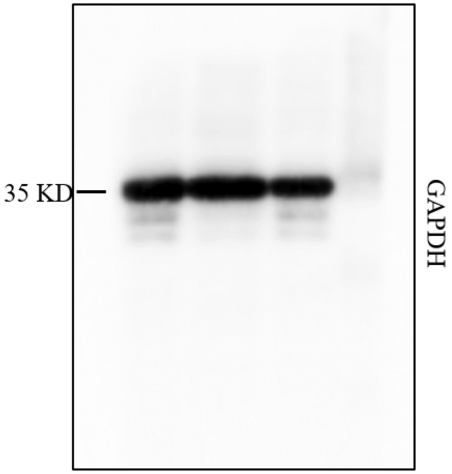

Fig. 5A

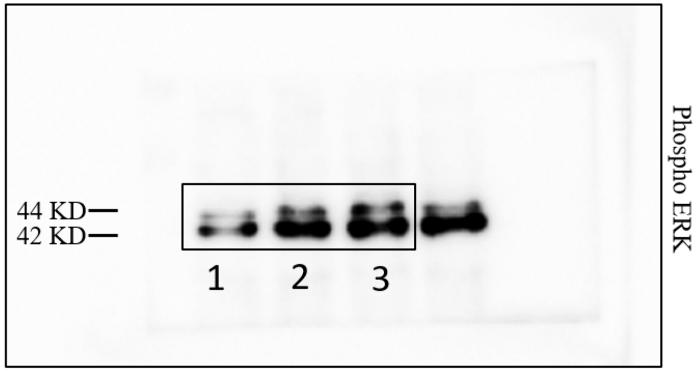

Fig. 5C (Lane 1,2,3 are on final figure)

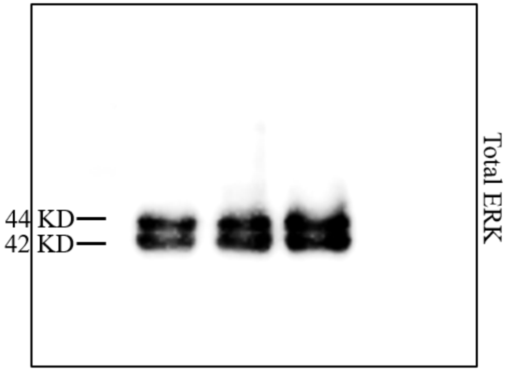

Fig. 5C

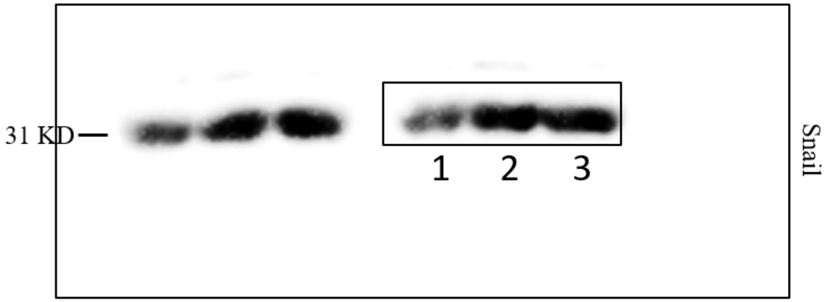

Fig. 5C (Lane 1,2,3 are on final figure)

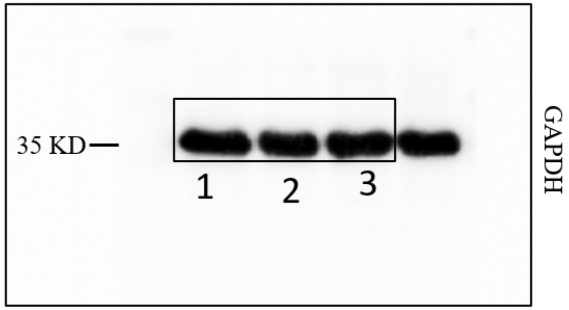

Fig. 5C (Lane 1,2,3 are on final figure)

ORIGINAL UNCROPPED FIGURE 7

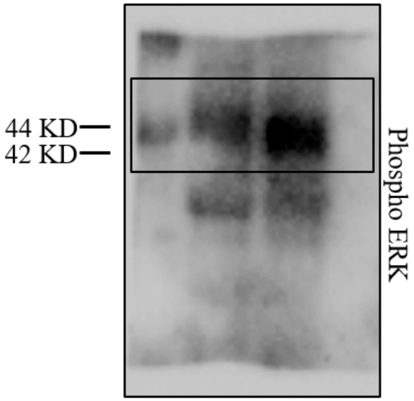

Fig. 7C

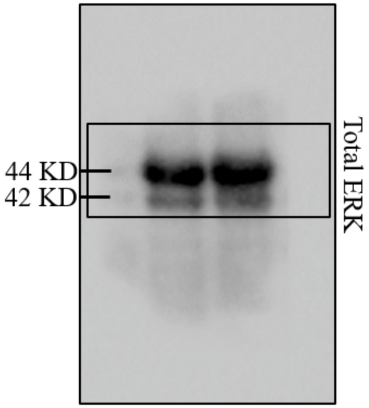

Fig. 7C

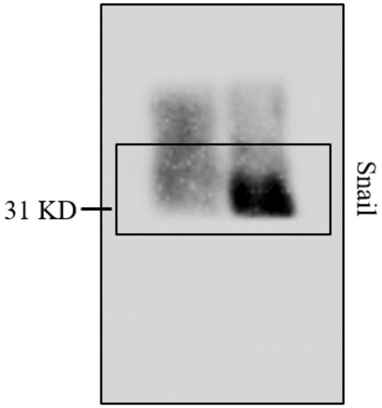

Fig. 7C

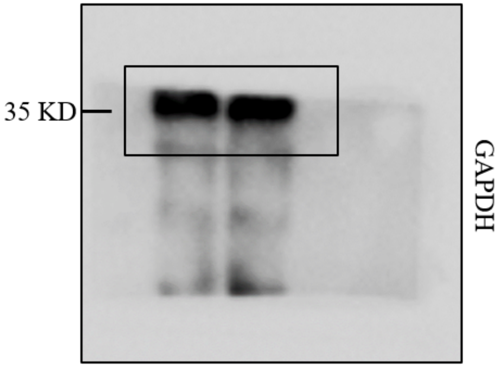

Fig. 7C

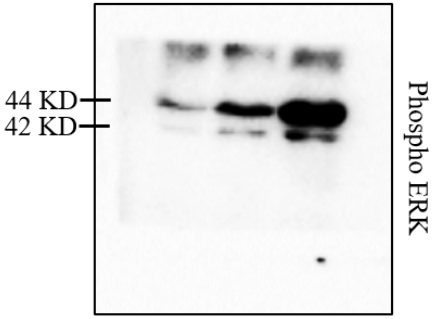

Fig. 7G

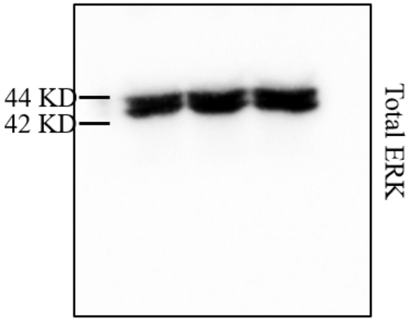

Fig. 7G

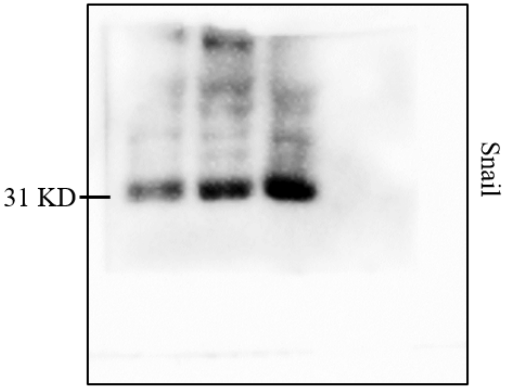

Fig. 7G

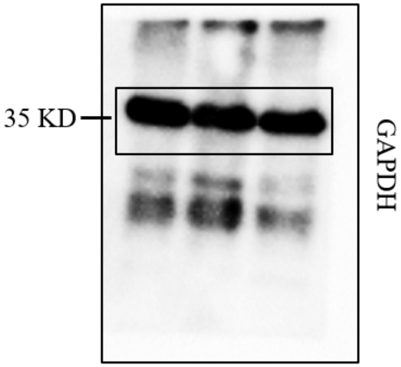

Fig. 7G

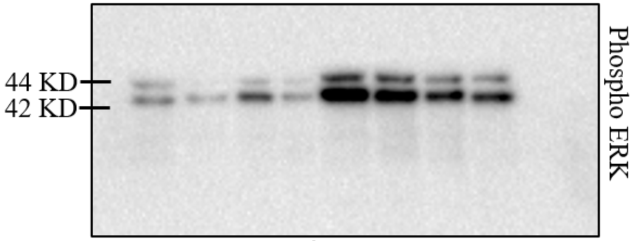

Fig. 7J

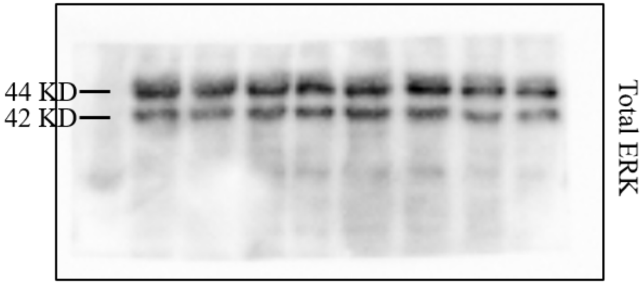

Fig. 7J

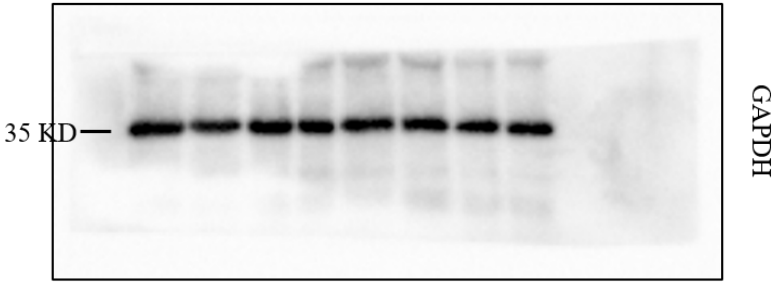

Fig. 7J

ORIGINAL UNCROPPED SUPPLEMENTARY FIGURE. 1

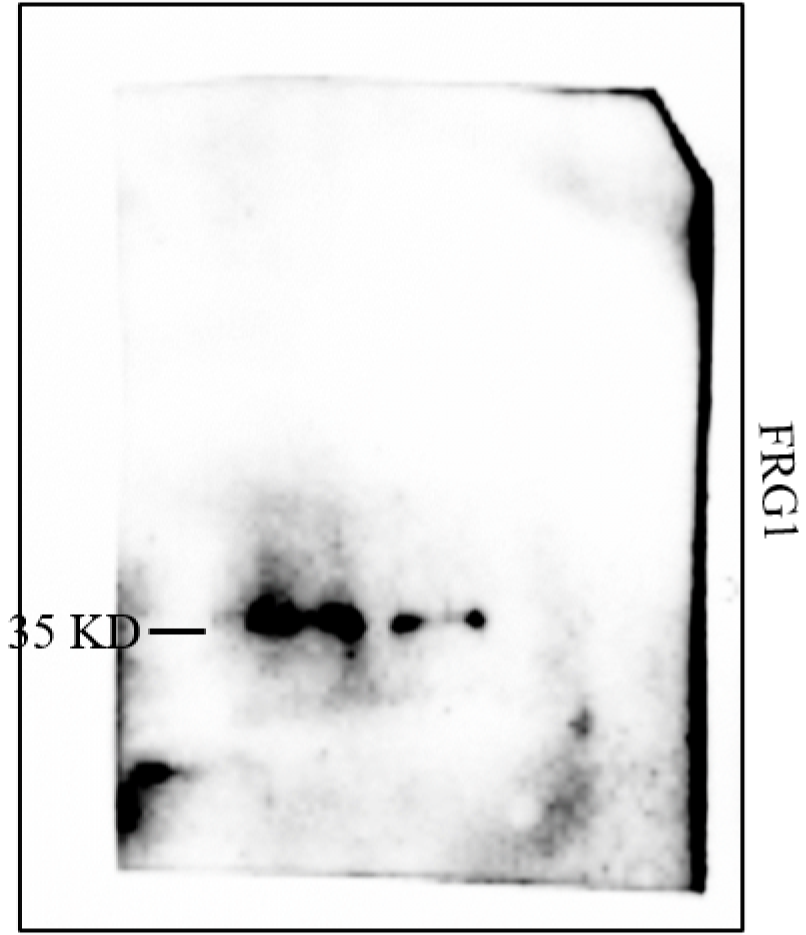

Supplementary Fig. 1A

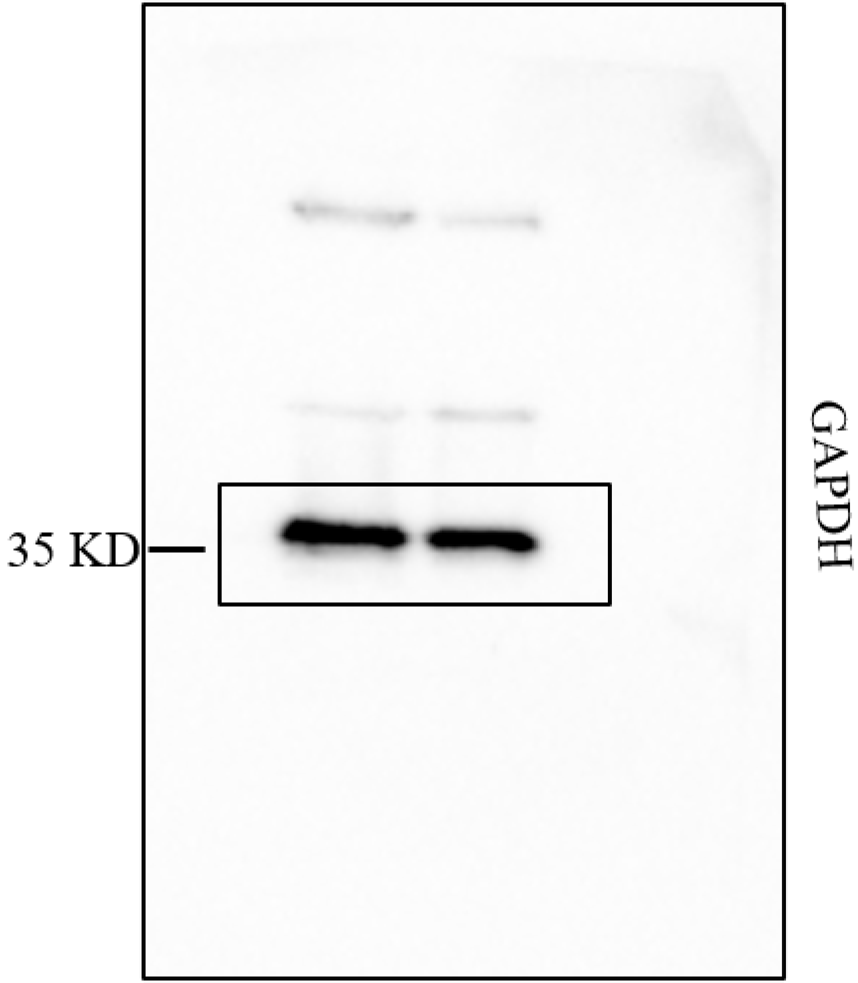

Supplementary Fig. 1A

ORIGINAL UNCROPPED SUPPLEMENTARY FIGURE. 1

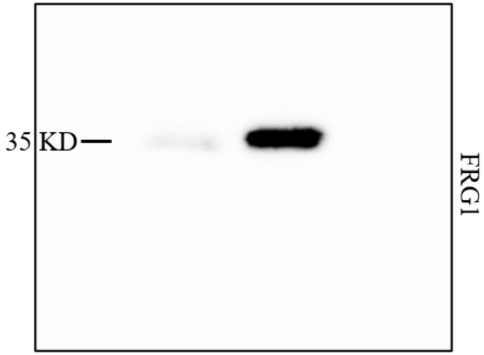

Supplementary Fig. 1D

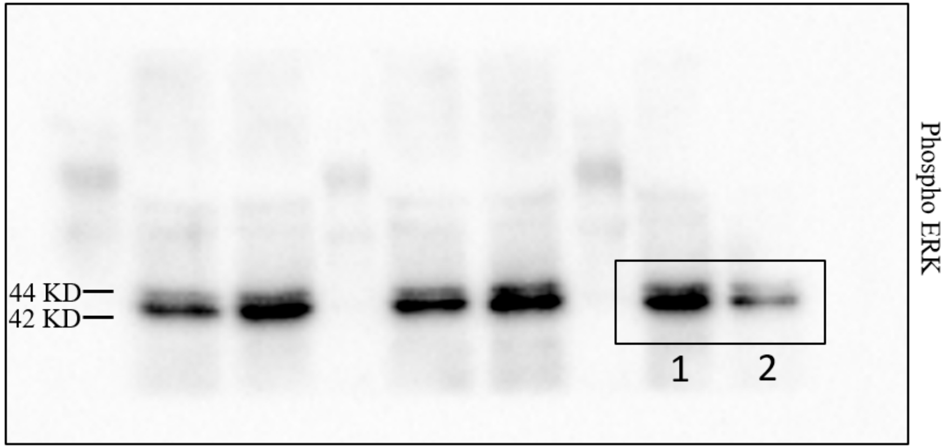

Supplementary Fig. 1D

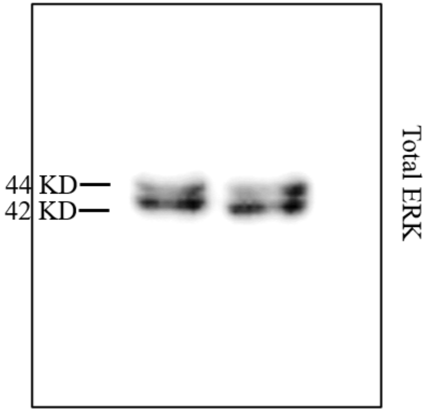

Supplementary Fig. 1D

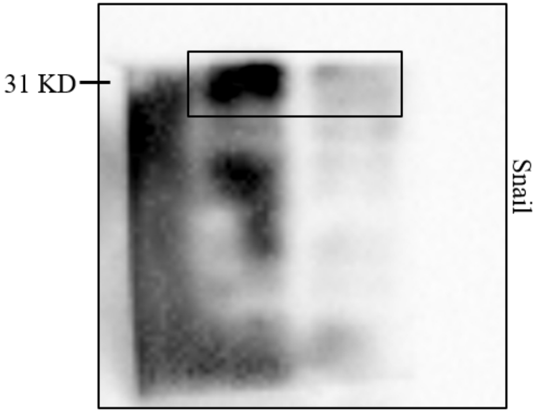

Supplementary Fig. 1D

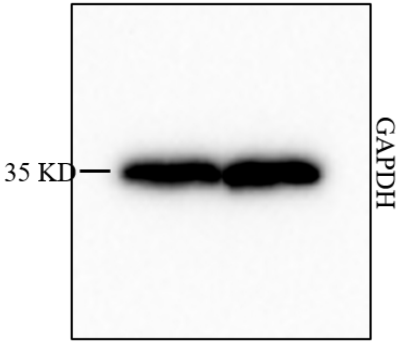

Supplementary Fig. 1D

ORIGINAL UNCROPPED SUPPLEMENTARY FIGURE. 2

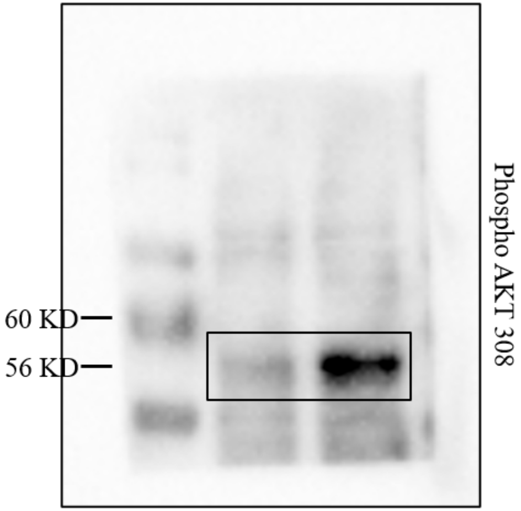

Supplementary Fig. 2A

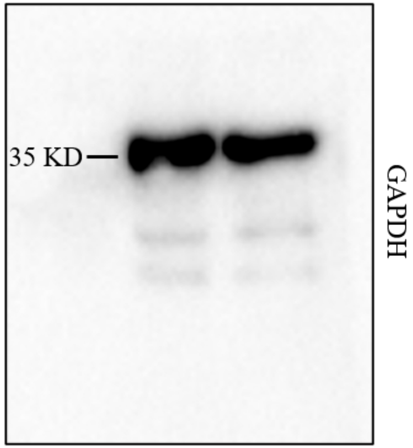

Supplementary Fig. 2A

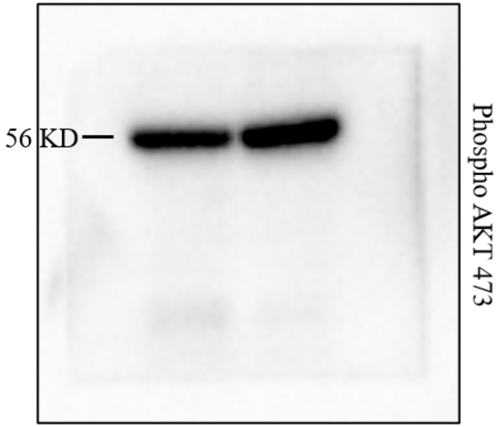

Supplementary Fig. 2B

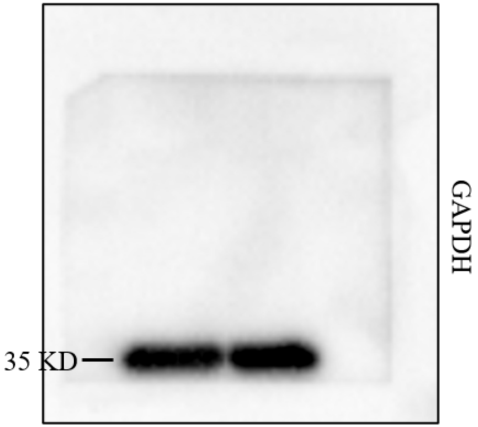

Supplementary Fig. 2B

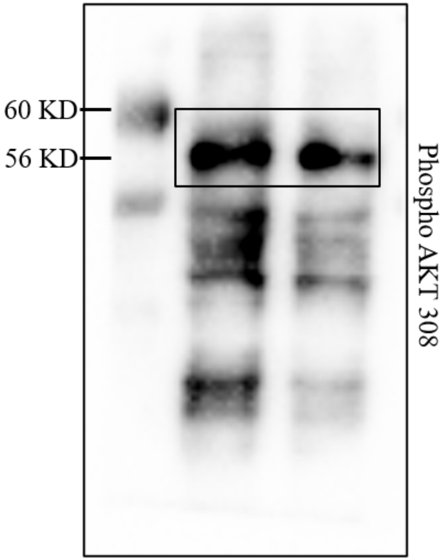

Supplementary Fig. 2C

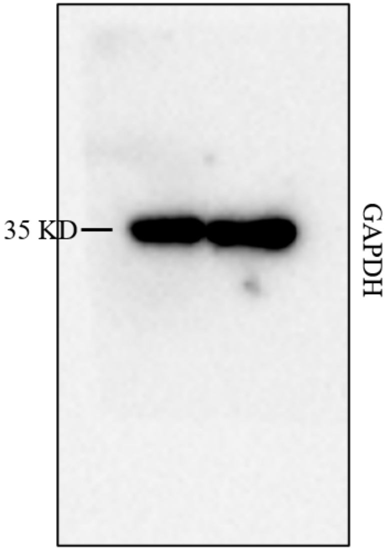

Supplementary Fig. 2C

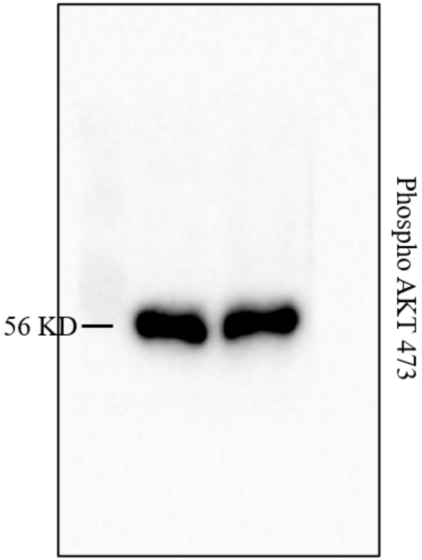

Supplementary Fig. 2D

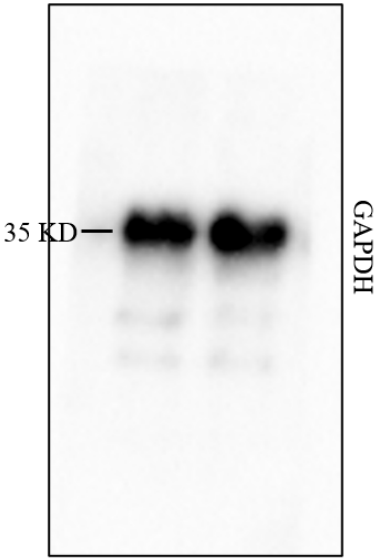

Supplementary Fig. 2D

ORIGINAL UNCROPPED SUPPLEMENTARY FIGURE. 2

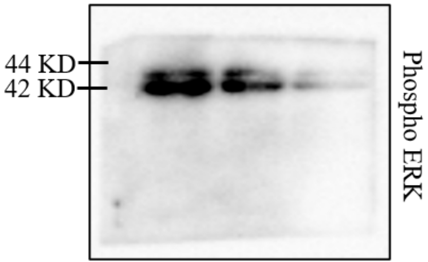

Supplementary Fig. 2E

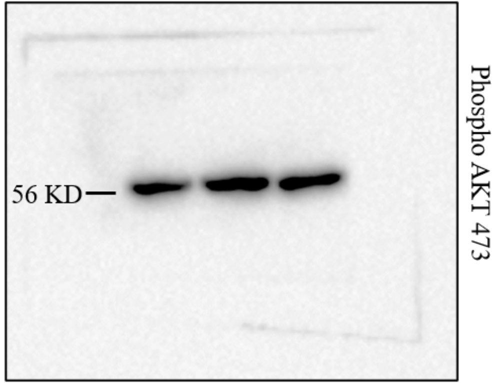

Supplementary Fig. 2E

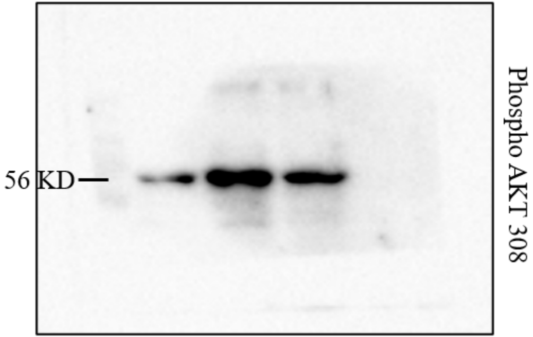

Supplementary Fig. 2E

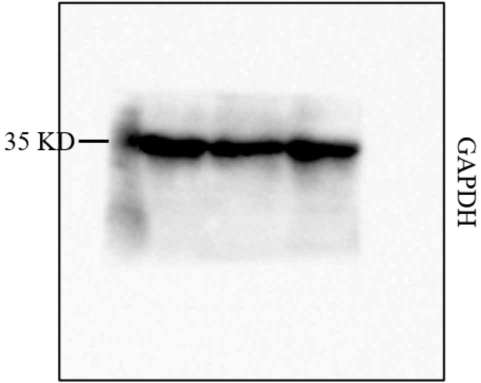

Supplementary Fig. 2E

ORIGINAL UNCROPPED SUPPLEMENTARY FIGURE. 3

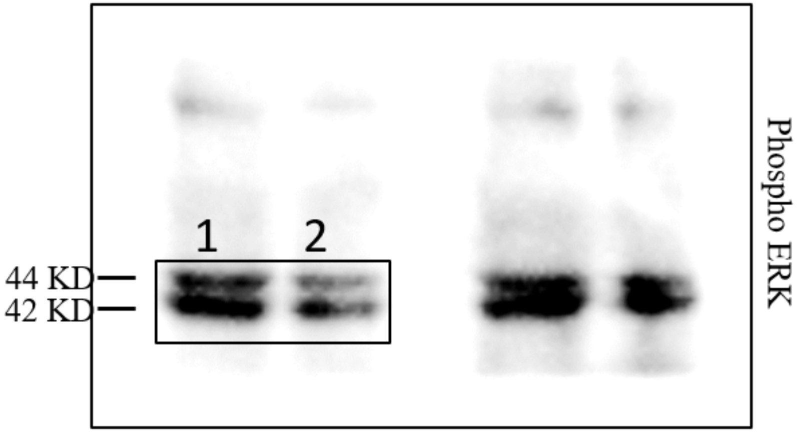

Supplementary Fig. 3C (Lane 1,2, are on final figure)

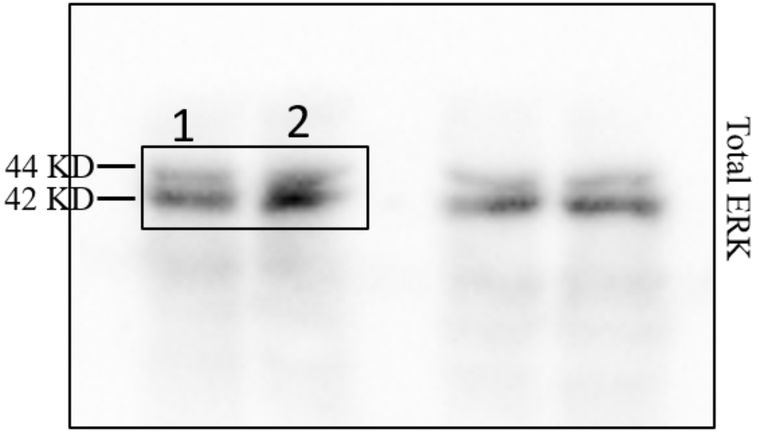

Supplementary Fig. 3C (Lane 1,2, are on final figure)

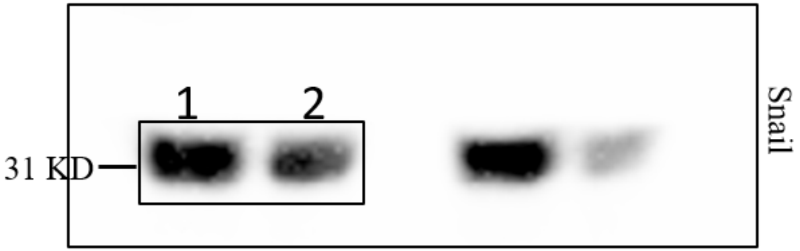

Supplementary Fig. 3C (Lane 1,2, are on final figure)

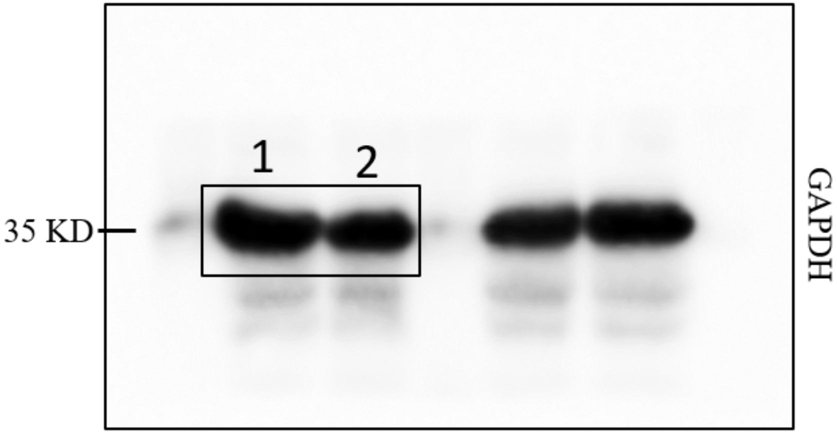

Supplementary Fig. 3C (Lane 1,2, are on final figure)

ORIGINAL UNCROPPED SUPPLEMENTARY FIGURE. 4

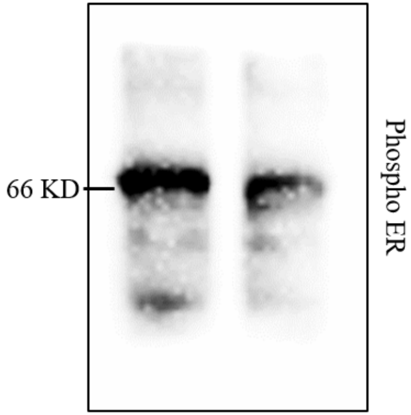

Supplementary Fig. 4B

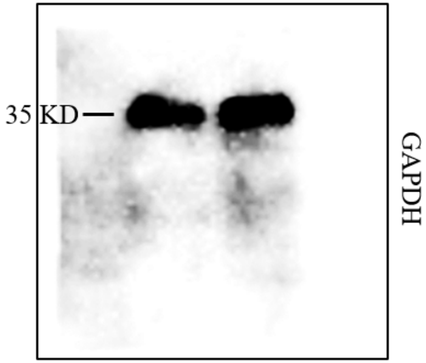

Supplementary Fig. 4B

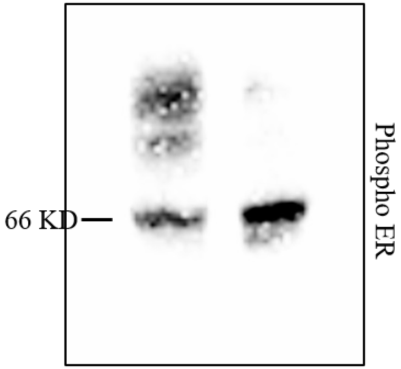

Supplementary Fig. 4C

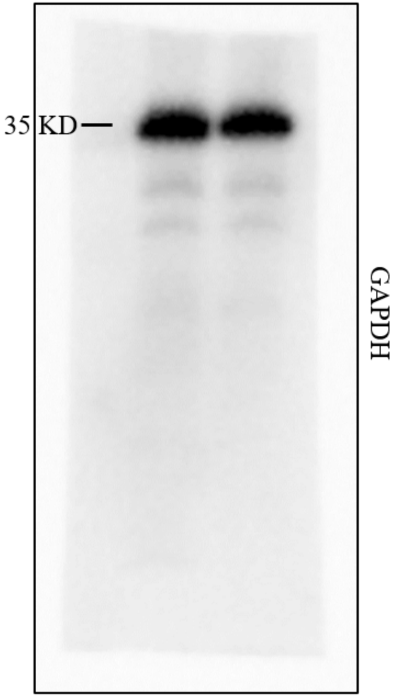

Supplementary Fig. 4C

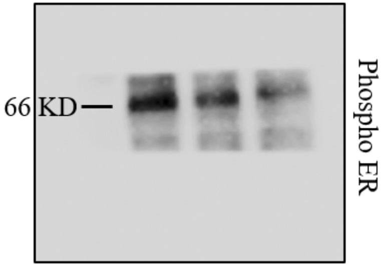

Supplementary Fig. 4D

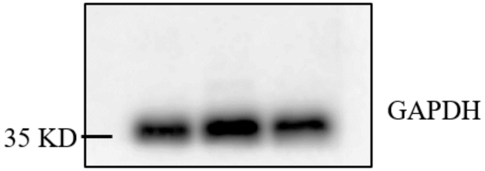

Supplementary Fig. 4D
